# MagNanoTrap Enrichment Empowers Ultra‐Sensitive Quantification of Mixed Nanoplastic Particles From Environmental Water Samples

**DOI:** 10.1002/anie.202522823

**Published:** 2026-04-05

**Authors:** Maochao Mao, Marian Bienstein, Francisca Contreras, Dong Wang, Lilin Feng, Ulrich Schwaneberg

**Affiliations:** ^1^ Institute of Biotechnology RWTH Aachen University Aachen Germany

**Keywords:** material‐binding peptide, nanoplastics, Py‐GC/MS, SPIONs, water remediation

## Abstract

The detection and quantification of nanoplastic particles (NPs) in environmental waters are crucial for monitoring their fate and assessing health impacts. However, the lack of sensitive and universal detection systems hinders effective regulation, as noted by the EU Commission. Here, we present the MagNanoTrap enrichment platform for NPs capture, which integrates a bifunctional peptide (LCI‐DZ‐MBP1) with Fe_3_O_4_ superparamagnetic nanoparticles (SPIONs). The LCI‐peptide binds to SPIONs, while the MBP1‐peptide serves as a general binder for common polymers such as polypropylene (PP), polyethylene (PE), polystyrene (PS), and polyethylene terephthalate (PET). MagNanoTrap exhibits a maximum adsorption capacity of 3.95 ± 0.14 g/g for PS‐COOH_500 nm_ NPs and, when coupled with pyrolysis–gas chromatography/mass spectrometry (Py‐GC/MS), enables reliable compositional analysis for mixed NPs and quantification down to 0.061 µg for PS. The achieved sensitivity ensures that a 1 L water sample is usually sufficient to detect and quantify NPs in environmental water samples. High salt ion concentrations promote hydrophobic interactions, enhancing NPs' binding affinity. The versatility of MagNanoTrap was demonstrated through successful enrichment and quantification of diverse NPs, including PP, PS, PE, PET, poly(methyl methacrylate), polycarbonate, nylon 6, and nylon 66, across seven environmental water matrices from river, lake, marine, and wastewater sources.

## Graphical abstract

Fe_3_O_4_ nanoparticles coated with the bifunctional peptide LCI‐DZ‐MBP1, where LCI binds the iron oxide surface and MBP1 acts as a universal plastic‐binding peptide, combined with pyrolysis‐gas chromatography/mass spectrometry (Py‐GC/MS), enable efficient enrichment, precise detection, and quantification of mixed plastics (PS, PE, PP, PET, PMMA, PC, Nylon 6, Nylon 66) from river, lake, sea, and wastewater sources.

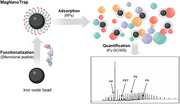

## 1 Introduction

Polymers such as polypropylene (PP), polystyrene (PS), polyethylene (PE), polyethylene terephthalate (PET), poly (methyl methacrylate) (PMMA), polycarbonate (PC), nylon 6, and nylon 66 were produced in 413.8 million metric tons in 2023 (Plastics—the fast Facts 2024 • Plastics Europe), due to their excellent properties in mechanical strength, ductility, and corrosion resistance, and so on. A responsible use of polymers requires the development of a sustainable circular polymer economy [1]. Policies, such as limits on virgin plastic production, reductions in unnecessary plastic use, and investments in waste and recycling management, will very likely lead to significant progress in both open‐ and closed‐loop polymer recycling. [2, 3] While a circular polymer economy is expected to reduce macroplastic leakage, repeated material processing, recycling, and extended product lifetimes may increase secondary micro‐ and nanoplastic (MP/NP) formation during use and reprocessing cycles. [4] For instance, out of 1 g of polyester textiles, up to 100 billion plastic nanoparticles within the size of 1 µm are released. [5, 6] Nowadays, MP/NPs are globally pervasive, contaminating environments from the atmosphere [7, 8] to water systems like rivers, [9, 10] lakes, [9, 10, 11] and oceans, [12] and can even be found in food (e.g., honey [13] and infant formula [14]) as well as bottled water [15] or beverages. [16] The four most commonly reported MP/NP contaminations are PP, PE, PS, and PET [17], with hydrophobic polymers particularly known for accumulating toxic hydrophobic compounds, such as polycyclic aromatic hydrocarbons (PAHs), plastic additives, and pharmaceuticals. [18] Recent studies have linked the accumulation of MP/NPs in plant roots to inhibiting plant growth and seed germination. [19, 20, 21] The ingestion of MP/NPs by animals has also been reported, [22, 23] with mussels, [24, 25] and zebrafish [26] being proposed as biomarkers for MP/NP contaminations. [25] Furthermore, reports indicate the accumulation of MP/NPs in human brains [27] and, particularly, NPs below 20 nm could cross the blood‐brain barrier, contributing to neurological disorders (e.g., amyotrophic lateral sclerosis [28] and Parkinson's disease [29].

In essence, to ensure environmental and human health, it is important to develop enrichment methodologies to identify and quantify MP/NPs in aqueous environments, beverages, and food products. [4] The analytical gaps to reliably quantify particles below a size of 5 mm have been acknowledged by the EU Commission as a main limitation in introducing regulations on MP/NP contaminations. [30, 31] To date, reported remediation technologies for the removal of MP/NPs comprise flotation systems based on bubble‐particle interactions, [32, 33] membrane‐based approaches leveraging the benefits of membrane cut‐off, [34, 35, 36, 37, 38] and physically or chemically surface‐functionalized materials driven by an adsorption process. [39, 40, 41, 42] However, only membrane‐based approaches have been reported as generally applicable for monitoring MP/NP contamination in environmental water samples. Specifically, these approaches have been implemented in cross‐flow systems requiring 50 L of environmental water samples [35, 36] and dead‐end systems processing 1 L samples. [37, 38] Both systems utilized polyethersulfone (PES) membranes with a 0.01 µm cutoff and were coupled with pyrolysis‐gas chromatography/mass spectrometry (Py‐GC/MS) for quantifying mixed plastics, including PP, PE, PS, PET, PMMA, PC, Nylon 6, and Nylon 66, achieving recovery exceeding 50%. [35, 36, 37, 38] Surface‐functionalized magnetic adsorbents, commonly based on Fe_2_O_3_/Fe_3_O_4_ that are often referred to as superparamagnetic iron oxide nanoparticles (SPIONs), comprise magnetic micro‐/nanorobots, [43, 44, 45] magnetic metal‐organic frameworks (MOFs), [46, 47] and chemically‐modified magnetic beads. [48, 49, 50] These systems have shown removal efficiencies above 80%, primarily for spiked PS and PMMA NPs, while the general applicability has not yet been investigated with environmental water samples.

Analytical methods are essential for the chemical identification and quantification of MP/NPs. Vibrational spectroscopy techniques such as Fourier‐transform infrared spectroscopy (FTIR) and Raman spectroscopy can only detect MPs larger than 1 µm, [51] while light scattering methodologies such as Nanoparticle tracking analysis (NTA) enable quantification but cannot distinguish between inorganic particles and organic MPs/NPs. [52] Py‐GC/MS is a promising methodology, not limited by particle size in terms of chemical detection, that can determine and quantify mixed plastics [53] and additives. [54] However, as a mass‐based thermal degradation technique, it does not provide information on particle size and morphology. Nevertheless, studies report a successful Py‐GC/MS application for detecting and quantifying mixed MP/NPs in environmental and biological samples, [27, 35, 36, 37, 38, 55, 56, 57] with detection limits down to 0.04 µg for PP and 0.07 µg for PE. [37]

The MBP technology was summarized, including limitations and application potential, in a recent comprehensive review [58] in which the MBPs were divided into naturally occurring binding peptides (nMBPs) and man‐made or engineered binding peptides (eMBPs). MBPs are typically smaller than 100 amino acids, [58] and can strongly bind to the surfaces of natural materials, such as leaves, [59, 60, 61] graphite, [62] and metals, [63, 64] as well as synthetic materials such as PP, [65] PS, [66] PET, [67] PLA, [68, 69] nylon 66, [70] and stainless steel. [71, 72] A well‐studied peptide is the liquid chromatography peak I (LCI) peptide, which consists of 47 amino acids and adopts a β‐sheet structure. [73] Another peptide, MBP1, contains 33 amino acids arranged in two parallel α‐helices, with roughly one‐third of its residues being arginine. [74] Interestingly, the incorporation of positively charged amino acids, such as arginine or lysine, has been reported to significantly improve binding strength to polymer surfaces, such as PP [75], PET [70], and polyamide. [70] MBP‐binding generally occurs under ambient temperatures in aqueous systems within a few minutes and could even withstand laundry at 60°C. [70, 76] Bifunctional peptides are usually generated by gene fusion of two MBPs with a stiff linker in between, which spatially separates both domains and thereby ensures their binding functionalities. [59, 77] Reported applications of bifunctional proteins comprise mainly plant health, [59, 60, 61] biocatalysis, [62, 71, 78] and medicine. [77]

**FIGURE 1:**
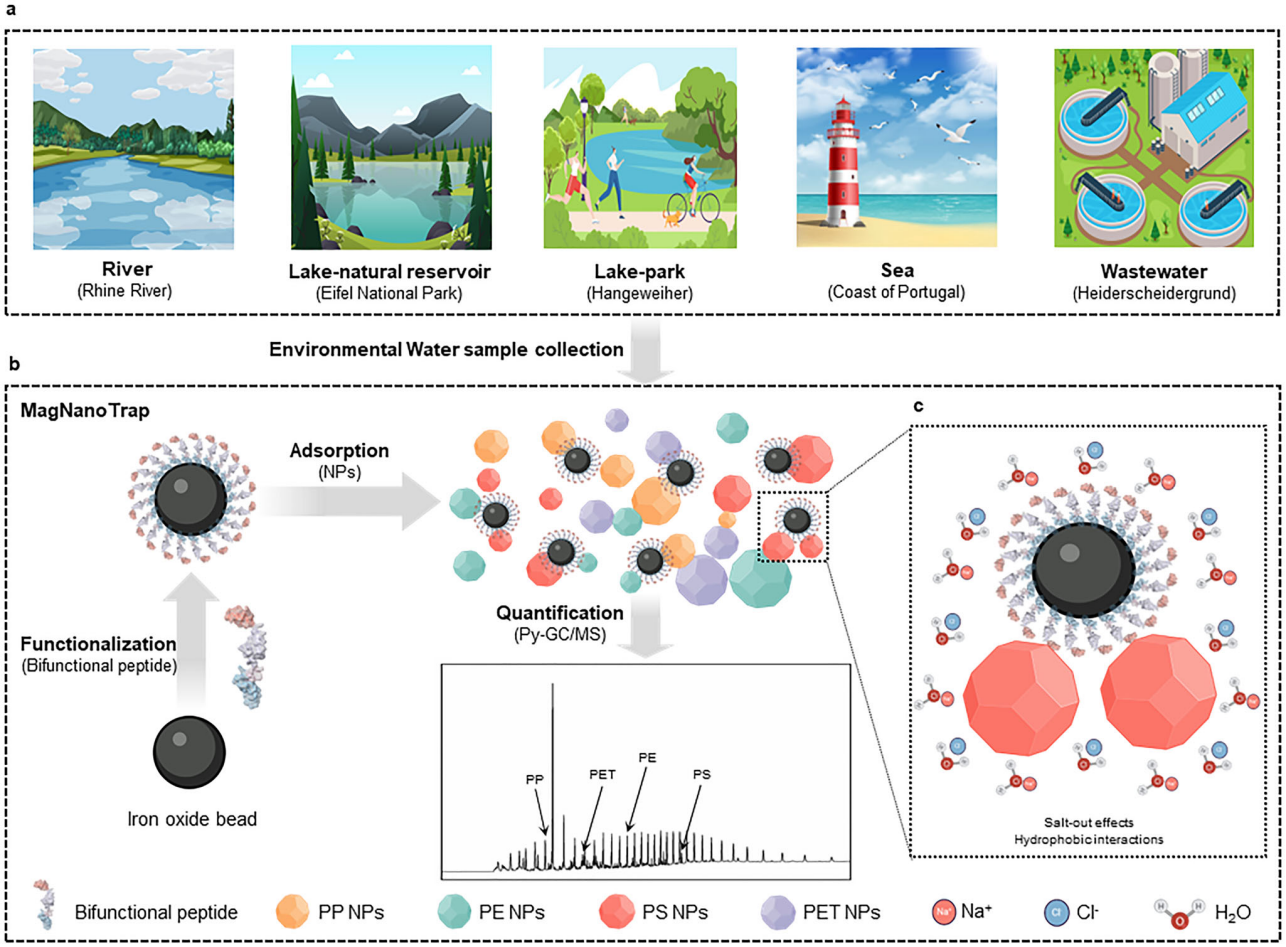
Overview of the MagNanoTrap monitoring platform for NPs enrichment and quantification in environmental water samples. (a) Origins of environmental water samples investigated in this study. (b) Scheme of the MagNanoTrap enrichment platform to determine and quantify NPs contamination in water samples with Py‐GC/MS. (c) Scheme of salt effect to facilitate the capture of NPs with MagNanoTrap beads. Protein models are visualized and coloured by ChimeraX 1.4. [79].

Here, we developed and validated the MagNanoTrap, termed the enrichment platform, based on a bifunctional peptide (LCI‐DZ‐MBP1) combined with SPIONs (Figure 1). The bifunctional peptide was designed to decorate SPIONs for NPs capture, with LCI binding to SPIONs and MBP‐1 acting as a general binder for PP, PE, PS, and PET. System validation included evaluating enrichment performance in 200 µL of spiked NPs deionized water, assessing the salt effect on PS NPs adsorption, and testing enrichment performance in 1 L of spiked NPs. MagNanoTrap enabled, in combination with the Py‐GC/MS method, to determine the composition of mixed NPs reliably and quantify NP amounts down to 0.061 µg for PS. The achieved sensitivity ensured that a 1 L water sample was often sufficient to detect MP/NP contamination in environmental water samples. The platform demonstrated broad applicability for various polymers, including PP, PE, PS, PET, PMMA, PC, Nylon 6, and Nylon 66, and was successfully tested on environmental water samples from rivers, lakes, seas, and wastewater sources.

## 2 Results and Discussion

### 2.1 SPIONs Functionalization

**FIGURE 2:**
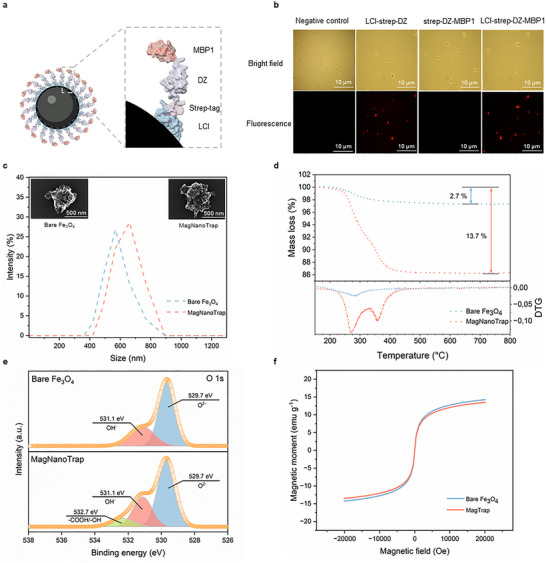
SPIONs functionalization with the bifunctional peptide LCI‐strep‐DZ‐MBP1. (a) Scheme illustrating decoration of a SPION by the bifunctional peptide LCI‐strep‐DZ‐MBP1. (b) Fluorescence microscope images demonstrating the functionalization of SPIONs with single peptides (LCI‐strep‐DZ and strep‐DZ‐MBP1) and the bifunctional peptide LCI‐strep‐DZ‐MBP1. (c) DLS measurements of bare (blue) and functionalized (orange) SPIONs, coupled with SEM images of bare (top left) and bifunctional peptide‐decorated SPIONs (top right). (d) TGA measurements of bare SPIONs (blue) and MagNanoTrap beads (orange). (e) XPS spectra (O 1s) of bare SPIONs (top) and MagNanoTrap (bottom). (f) Magnetization of bare SPIONs (blue) and MagNanoTrap (orange). Protein models are visualized and coloured by ChimeraX 1.4 [79].

The successful decoration of SPIONs (Fe_3_O_4_) with the bifunctional peptide LCI‐DZ‐MBP1, along with the characterization of the physical properties of both bare SPIONs and peptide‐decorated SPIONs, is shown in Figure 2. The SPIONs decorated with bifunctional peptides are subsequently referred to as MagNanoTrap.

Bifunctional peptide decoration of SPIONs was simply achieved by suspending SPIONs in a bifunctional peptide solution at room temperature, followed by a simple washing step with water after a 1‐min incubation (Figure 2a). The binding was demonstrated by employing a fluorescence‐labeled streptavidin protein, which specifically binds to the strep‐tag present in the fusion proteins LCI‐strep‐DZ, strep‐DZ‐MBP1, and LCI‐strep‐DZ‐MBP1. Fluorescent labeling enables visualization of the binding properties on both the SPION surface and the plastic surface. LCI, in the LCI‐strep‐DZ fusion protein, binds to SPIONs, whereas MBP1 (strep‐DZ‐MBP1) did not yield a detectable binding signal (Figure 2b). Further binding studies revealed that MBP1 is a universal binder to a wide range of synthetic polymers, including PP, PE, PS, and PET (Figure S1). The latter results show that the combination of LCI and MBP1 is an excellent bifunctional peptide design for MagNanoTrap development. Additionally, flow cytometry analysis confirmed that 98% of SPIONs were successfully decorated with the bifunctional peptide LCI‐DZ‐MBP1 (Figure S2). In Figure 2c, the morphology and size dispersity were determined by scanning electron microscopy (SEM) and dynamic light scattering (DLS). DLS analysis confirmed that no aggregation of MagNanoTrap beads occurred and, as expected, showed an increased hydrodynamic radius (Figure 2c). SEM images showed that SPION morphology was preserved by decoration with a nanometer‐sized peptide layer (Figures 2c and S3). Thermogravimetric analysis (TGA) confirmed LCI‐DZ‐MBP1 decoration of SPIONs, as indicated by an increase in mass loss from 2.7% to 13.7%. Additionally, a new peak appeared in the derivative curve at 350°C, corresponding to the thermal degradation of the peptide layer (Figure 2d). The presence of peptides on the surface of SPIONs was further confirmed by electrospray ionization coupled with mass spectroscopy (ESI‐MS), in which the detected molecular weight closely aligns with the size (17.49 kDa) determined by SDS‐PAGE analysis (Figures S5 and S6). X‐ray photoelectron spectroscopy (XPS) also confirmed the successful binding of LCI‐DZ‐MBP1 to SPIONs. In detail, the O 1s spectrum of MagNanoTrap displayed an additional component at ∼532.7 eV compared to bare SPIONs. This peak is attributed to peptide‐associated oxygen species, likely originating from side‐chain functional groups (─COOH and ─OH; Figure 2e). FTIR further confirmed the characteristic of the N─H bond (1632 cm^−1^) that peptide LCI‐DZ‐MBP1 decorates SPIONs (Figure S7). Vibrating sample magnetometer (VSM) analysis revealed that LCI‐DZ‐MBP1 nanocoating had a negligible effect on the magnetic moment and superparamagnetic characteristics of the SPIONs (Figure 2f).

The aforementioned characterization indicates a successful decoration of SPIONs with the bifunctional peptide LCI‐DZ‐MBP1, resulting in the production of MagNanoTrap beads, which will be utilized in subsequent steps for the enrichment of NPs in water samples.

### 2.2 Enrichment Performance on a 200 µL Scale

**FIGURE 3:**
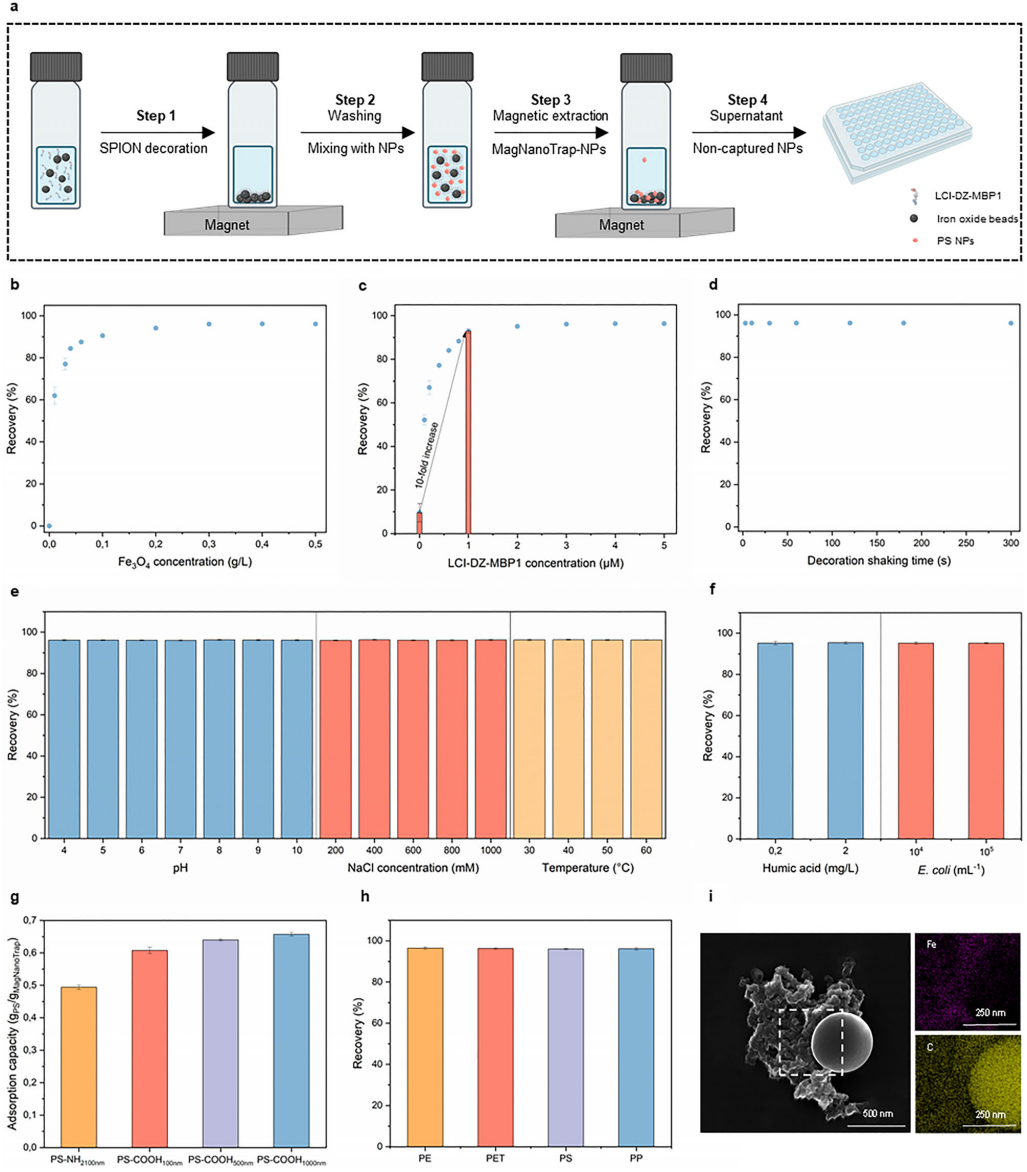
Adsorption efficiency of MagNanoTrap for NPs. (a) Scheme of the four‐step workflow developed for assessing the enrichment performance of MagNanoTrap technology on a 200 µL scale. In step 1, SPIONs were decorated with LCI‐DZ‐MBP1 in 50 mM Bicine, pH 9; in step 2, the MagNanoTrap beads were washed with water, followed by mixing with NPs in the presence of 150 mM NaCl; in step 3, the MagNanoTrap‐NPs complex was extracted by a magnet; and in step 4, the supernatant containing non‐captured NPs was taken for absorbance measurement. The recovery of PS‐COOH_500 nm_ was determined at (b) increasing MagNanoTrap concentrations, (c) increasing LCI‐DZ‐MBP1 peptide concentrations, and (d) different incubation times of SPIONs and LCI‐DZ‐MBP1 peptide for SPIONs functionalization, *n* = 3, mean ± SD. (e) Stability of MagNanoTrap beads under various pH (4 to 10), ionic strength (200 mM to 1 M NaCl), and temperature (30°C to 60°C), *n* = 3, mean ± SD. (f) Recovery of PS‐COOH_500 nm_ NPs in the presence of humic acid (0.2 and 2 mg/L) and *E. coli* (104 and 105/mL) with MagNanoTrap quantified by Py‐GC/MS, *n* = 3, mean ± SD. (g) Adsorption capacity of positively (PS‐NH_2–100 nm_) and negatively (PS‐COOH_100 nm_, PS‐COOH_500 nm_, and PS‐COOH_1000 nm_) charged NPs with MagNanoTrap, *n* = 3, mean ± SD. (h) Recovery of 0.2 g/L PE, PET, PS, and PP NPs with MagNanoTrap quantified by Py‐GC/MS, *n* = 3, mean ± SD. (i) SEM coupled with EDXS images showing PS COOH_500 nm_ NP was captured by MagNanoTrap beads, in which purple represents Fe elements in SPIONs (top right), and yellow represents C elements in PS NPs and MagNanoTrap beads (bottom right).

Enrichment experiments with PS, PP, PE, and PET NPs were performed on a 200 µL scale to evaluate the performance and general applicability of the MagNanoTrap enrichment technology. Figure 3a shows the four‐step workflow developed for assessing the enrichment performance of scalable MagNanoTrap technology, incorporating absorbance measurement for quantification of enrichment performance (Table S1). Enrichment optimization comprised determining the optimal SPION concentration (Figure 3b), the required LCI‐DZ‐MBP1 concentration for SPION decoration (Figure 3c), the minimal incubation time for efficient LCI‐DZ‐MBP1 decoration of SPIONs (Figure 3d), and the ideal NaCl concentration (Figure S8). In all experiments, 5 µM of LCI‐DZ‐MBP1 and 1 min decoration time were used to ensure a saturated decoration of SPIONs, while a 20‐min shaking time was employed for saturated PS‐COOH_500 nm_ NPs capture, with 150 mM NaCl supplemented in Step 2, unless otherwise specified. In detail, a concentration of 0.3 g/L of decorated SPIONs effectively recovered 0.2 g/L of PS‐COOH_500 nm_ NPs, achieving a recovery of 96.1% ± 0.3% (Figure 3b). Additionally, 1 µM of peptide was adequate to fully decorate 0.3 g/L of SPIONs, with LCI‐DZ‐MBP1 functionalization significantly enhancing NPs capture, increasing the recovery ratio by 10‐fold compared to bare SPIONs (Figure 3c). Notably, as shown in Figure 3d, just 3 s of shaking were sufficient for SPION decoration. Interestingly, the enrichment only occurred with the addition of NaCl, with 150 mM of NaCl reaching the saturated recovery (Figure S8). To evaluate the stability of the peptide–SPION conjugate under varying conditions, the modified SPIONs were pre‐incubated for 2 h at different pH values, ionic strengths, and temperatures prior to performing enrichment experiments under the optimized conditions (Figure 3e). As shown in Figure 3e, the enrichment efficiency remained essentially unchanged across all tested conditions. The recovery of PS‐COOH_500 nm_ NPs was consistently maintained at approximately 96%, demonstrating that the peptide–SPION conjugate exhibits strong stability and robust binding performance. To assess enrichment performance under realistic water‐matrix conditions, the enrichment experiments were conducted in the presence of natural organic matter (NOM) and suspended solids (Figure 3f). Humic acid was selected as a representative NOM component at concentrations of 200 µg/L and 2 mg/L, which fall within the typical range reported for natural waters. [80] *Escherichia coli* (*E. coli*) was employed as a model biological suspended solid at 10^4^ and 10^5^ cells/mL to simulate environmentally relevant particulate loadings. [81] As shown in Figure 3f, the enrichment efficiency for PS‐COOH_500 nm_ NPs remained nearly constant at approximately 95% under all tested conditions. These results suggest that no significant peptide desorption from the SPION surface or SPION oxidation occurred during the enrichment process, supporting the robustness and applicability of MagNanoTrap for NPs capture in complex water matrices.

The general applicability was investigated by applying the developed MagNanoTrap enrichment platform to PS NPs functionalized with amino groups (PS‐NH_2_, pKa 9–10) and carboxylate groups (PS‐COOH, pKa 4.75). Experiments were performed at pH 7, where PS‐NH_2_ NPs are positively charged, while PS‐COOH NPs are negatively charged (Figure S9). The adsorption capacities were determined as 494.3 ± 7.0 mg/g for PS‐NH_2 100 nm_, 607.6 ± 9.5 mg/g for PS‐COOH_100 nm_, 639.8 ± 3.2 mg/g for PS‐COOH_500 nm_, and 657.5 ± 6.0 mg/g for PS‐COOH_1000 nm_ (Figure 3g). A general trend was observed, where increasing PS‐COOH NP size (100 nm, 500 nm, and 1000 nm) resulted in higher adsorption capacities. Further validation of the enrichment efficiency was performed using three additional NP types: PP, PE, and PET. These self‐made NPs, prepared by emulsification‐solvent evaporation [82] and nanoprecipitation [83] methods, were characterized as negatively charged based on zeta potential measurements. The size distribution and morphology were analyzed using DLS and SEM, respectively, showing that the self‐made PP, PE, and PET particles are in nano‐size (Figures S9 and S10). After mixing with the MagNanoTrap beads and applying magnetic separation, the supernatants of all tested NP suspensions became completely clear. The corresponding recovery rates were 96.5%, 96.3%, 96.1%, and 96.2% for PE, PET, PS, and PP, respectively, confirming the efficient removal of nanoparticles from the suspension (Figures 3h and S11, and Supplementary Video). Additionally, SEM/EDXS images of MagNanoTrap after capturing PS‐COOH_500 nm_ NPs provided further evidence of successful SPION functionalization and NPs adsorption (Figure 3i). These results collectively validate the bio‐functionalized MagNanoTrap platform as a universal NPs enrichment platform.

### 2.3 Cations Effect

**FIGURE 4:**
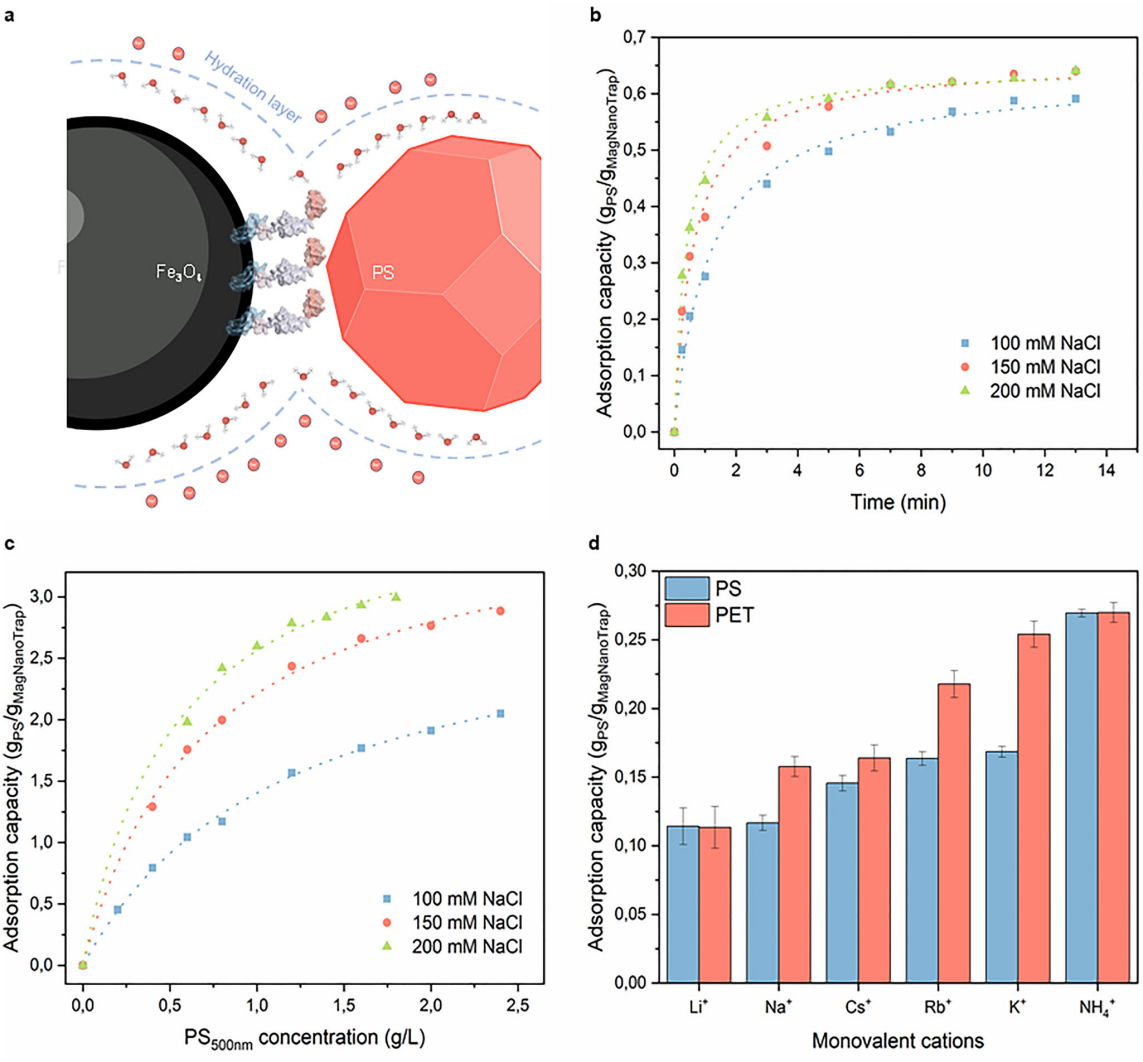
NaCl concentration and cation effects on NPs adsorption. (a) Scheme illustrating the NaCl effects on NPs adsorption by MagNanoTrap beads. (b) Adsorption kinetics of MagNanoTrap platform for PS‐COOH_500 nm_ NPs at different salt concentrations (100, 150, and 200 mM) fitted with the pseudo‐second‐order model, *n* = 3, mean ± SD. (c) Adsorption isotherm of MagNanoTrap platform for PS‐COOH_500 nm_ NPs at different salt concentrations (100, 150, and 200 mM) fitted with the Langmuir model, *n* = 3, mean ± SD. (d) Adsorption capacity of PS‐COOH_500 nm_ NPs and self‐made PET NPs by MagNanoTrap platform in the presence of 50 mM monovalent cations (Li^+^, Na^+^, Cs^+^, Rb^+^, K^+^, and NH_4_^+^) during a 30‐s reaction, *n* = 3, mean ± SD. Protein models are visualized and coloured by ChimeraX 1.4. [79].

Salt concentrations are known to influence the binding properties of peptides to hydrophobic interaction chromatography (HIC). [84] In order to develop a generally applicable MagNanoTrap enrichment technology, the effect of salt on the binding properties of the exposed peptide MBP1 to PS NPs was investigated. Salt NaCl was selected to ensure that the MagNanoTrap platform remains functional in seawater environments. During the adsorption process, NaCl ions likely competed with the interactions between water molecules and the charged groups on the surfaces of MagNanoTrap and NPs, weakening the hydration layer, which in turn made the hydrophobic regions more exposed [85] (Figure 4a). To gain deeper insight into the role of NaCl in NPs enrichment, NaCl effects on adsorption kinetics and adsorption isotherms were investigated. Kinetic studies showed rapid adsorption of 0.2 g/L PS‐COOH_500 nm_ NPs by 0.3 g/L of MagNanoTrap beads, achieving adsorption saturation within a 13 min reaction (Figure 4b). The experimental data aligned with a pseudo‐second‐order adsorption kinetic model, [86] with the adsorption rate constant, 𝑘_2_, increasing significantly as the salt concentration rose. Specifically, 𝑘_2_ values increased from 1.36 ± 0.16 g/(g·min) at 100 mM to 4.04 ± 0.29 g/(g·min) at 200 mM, demonstrating the critical role of NaCl in accelerating the reaction rate. As expected, the adsorption uptake capacity at equilibrium, *q*_e_, remained relatively consistent, with values of 0.63 ± 0.01 g/g, 0.66 ± 0.01 g/g, and 0.64 ± 0.01 g/g for 100 mM, 150 mM, and 200 mM NaCl, respectively (Table S2). To determine how the adsorption affinity and maximum adsorption capacity of MagNanoTrap against PS‐COOH_500 nm_ NPs are affected by NaCl concentration, the adsorption isotherm was investigated and fitted to both the Langmuir and the Freundlich models [87] (Figure 4c and Table S3). The adsorption data showed a good fit to the Langmuir model, with a correlation coefficient >0.99, suggesting monolayer adsorption and a uniform adsorption surface of the material. [87] The increase of Langmuir constant, *K*_L_, with NaCl concentration, from 0.85 ± 0.05 L/g at 100 mM to 1.85 ± 0.21 L/g at 200 mM, implied enhanced adsorption affinity due to the presence of NaCl. The maximum adsorption capacities, *q*_m_, calculated by the model were 3.05 ± 0.08 g/g, 3.80 ± 0.09 g/g, and 3.95 ± 0.14 g/g at 100, 150, and 200 mM of NaCl, respectively, outperforming many previously reported adsorbent materials, such as biomass fibrous foam (444.6 ± 22.3 mg/g) [40] and SPIONs‐PAC_18_ (0.69 ± 0.26 g/g) [48] for PS NPs. Interestingly, at the same NaCl concentration, the adsorption capacity was lower for smaller PS‐COOH NPs compared to larger NPs, showing adsorption of 517.6 ± 10.6 mg/g for PS‐COOH_100 nm_, 591.2 ± 2.2 mg/g for PS‐COOH_500 nm_, and 649.4 ± 5.2 mg/g for PS‐COOH_500 nm_ at 100 mM NaCl, since smaller NPs with larger surface area require more NaCl to neutralize the surface charges (Figure S12). To further validate the hypothesis, the enrichment with 50 mM of various monovalent cations was performed over a 30‐s reaction for PS‐COOH_500 nm_ and self‐made PET NPs. The adsorption capacity of MagNanoTrap mediated by different cations followed the order NH_4_^+^ > K^+^ > Rb^+^ > Cs^+^ > Na^+^ > Li^+^, consistent with the Hofmeister series, which illustrated the salting‐out ability of the cations [88] (Figure 4d). The XPS spectra of MagNanoTrap before and after PS nanoparticle capture were analyzed to provide further molecular‐level insight into the peptide–polymer interaction (Figures S13 and S14). In the peptide–Fe_3_O_4_ conjugate, the O 1s spectrum exhibits a component at ∼531.1 eV corresponding to peptide‐associated oxygen, primarily assigned to amide carbonyl groups. After PS capture, this component shifts to ∼531.8 eV. In addition, the higher binding energy component at ∼532.7 eV, attributed to oxygen species from peptide side‐chain functional groups (e.g., ─COOH and ─OH), also shifts to ∼533.2 eV. A concurrent positive shift of approximately 0.7 eV is observed in the N 1s peak. The simultaneous upward shifts in both amide nitrogen and carbonyl/side‐chain oxygen components indicate a change in the electronic environment of peptide functional groups upon interaction with PS nanoparticles. These increases in binding energy suggest reduced electronic shielding of peptide amide functionalities, consistent with altered interfacial solvation and changes in the local dielectric environment associated with hydrophobic association. This interpretation aligns with the observed salt‐enhanced adsorption behavior, in which increasing ionic strength weakens the hydration layer, exposing the hydrophobic surface for interaction. The effect of divalent and trivalent cations at varying salt concentrations was further examined (Figure S15). Following Lewis acid strength, the effectiveness of cations in promoting adsorption followed the order Al^3^^+^ > Mg^2^^+^ > Na^+^. [89] With increasing Lewis acidity and valency, a lower salt concentration was required to achieve the same adsorption capacity. This behavior suggests that multivalent cations might facilitate capture through Lewis acid–base interactions, acting as coordination bridges between functional groups on the MagNanoTrap surface and the nanoplastic particles.

In order to assess the applicability of the MagNanoTrap enrichment technology in environmental water samples containing heavy metals, enrichment experiments were performed using Cd^2^^+^, Zn^2^^+^, Cu^2^^+^, and Ni^2^^+^, instead of NaCl. The results demonstrated a recovery exceeding 99% for PS‐COOH_500 nm_ NPs, confirming that the presence of heavy metals does not interfere with the enrichment process (Figure S16). Using Zn^2^^+^ as a model, the adsorption capabilities of MagNanoTrap beads and PS‐COOH_500 nm_ NPs were evaluated, both individually and in combination, for adsorbing heavy metals. At a Zn^2^^+^ concentration of 20 mM, MagNanoTrap beads (0.3 g/L) showed an uptake of 1292.3 ± 55.5 µM of Zn^2^^+^, while PS‐COOH_500 nm_ NPs (0.2 g/L) alone, adsorbed 538.5 ± 40.7 µM of Zn^2^^+^. Interestingly, when PS‐COOH_500 nm_ NPs were incubated in combination with MagNanoTrap beads, the total Zn^2^^+^ adsorption reached only 1456.4 ± 38.7 µM, less than the sum of the individual adsorption capacities, suggesting that Zn^2^^+^ ions mediate the interaction between PS‐COOH_500 nm_ NPs and MagNanoTrap beads (Figures S17 and S18). The aforementioned results showed that NaCl supplementation enhances MBP1 binding to plastic surfaces, and heavy metals do not influence the enrichment process. It highlights the general applicability of the MagNanoTrap platform, which can be utilized in all kinds of natural aqueous environments, such as lakes, rivers, wastewater, and seawaters, as NaCl can always be added.

### 2.4 Enrichment Performance on a 1 L Scale Characterized by Py‐GC/MS

**FIGURE 5:**
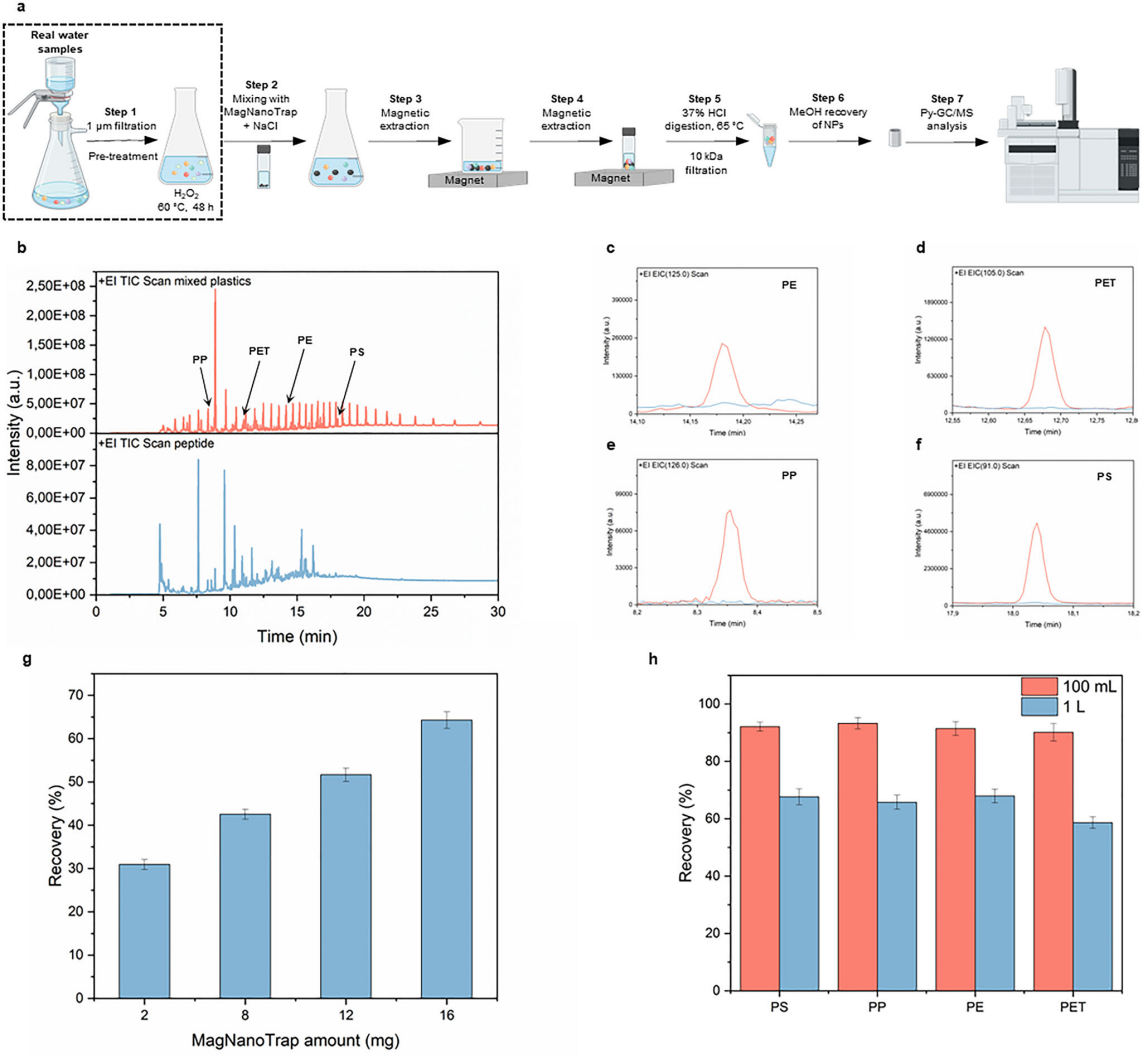
1 L‐scale enrichment characterized by Py‐GC/MS. (a) Scheme of the seven‐step workflow developed for assessing the enrichment performance of MagNanoTrap technology on a 1 L scale. In step 1, 1 µm filtration and H_2_O_2_ digestion were done to remove the large aggregates and organic matter; in step 2, MagNanoTrap beads were added to the pretreated environmental water samples with the supplementation of NaCl; in steps 3 and 4, MagNanoTrap‐NPs complex was extracted by a magnet to reduce the volume; in step 5, MagNanoTrap‐NPs complex was digested by 37% HCl at 65°C, followed by filtration with 10 kDa centrifugal filters; in step 6, the complex was recovered with methanol and transferred to Py‐GC/MS sampling cups; and in step 7, NP quantification was done by Py‐GC/MS. (b) Chromatograms of the mixed plastics containing PP, PE, PET, PS, PMMA, PC, PVC, nylon 6, nylon 66, ABS, and SBR (top) and the peptide LCI‐DZ‐MBP1 (bottom). Chromatograms with an extracted m/z of, (c) PE (m/z 125), (d) PET (m/z 105), (e) PP (m/z 126), and (f) PS (m/z 91) from both mixed plastics and peptides. (g) Recovery of PS‐COOH_500 nm_ NPs (5 µg spiked in 1 L deionized water) at different amounts of MagNanoTrap beads, with 1 M NaCl and 2 h shaking, *n* = 3, mean ± SD. (h) Recovery of mixed NPs (PS, PP, PE, and PET, each spiked with 1 µg in 100 mL and 1 L of deionized water) by 16 mg of MagNanoTrap beads, *n* = 3, mean ± SD.

In this section, we aim to prove that the NaCl‐facilitated enrichment protocol can be scaled up to a 1 L volume by investigating the recovery of spiked mixed PP, PE, PET, and PS NPs in deionized water. Figure 5a shows the seven‐step workflow developed for assessing the enrichment performance of MagNanoTrap technology on a 1 L scale, incorporating Py‐GC/MS analysis for the detection and quantification of mixed plastics (Table S4). To ensure accurate plastic quantification, extracted peaks from the mixed plastics, including PP, PE, PET, PS, PMMA, PC, PVC, nylon 6, nylon 66, ABS, and SBR, were compared against those from the bifunctional peptide LCI‐DZ‐MBP1 (Figure 5b). The specific molecular markers used for PP, PE, PET, and PS quantification were 2,4,6‐dimethyl‐1‐heptene (m/z 126), 1‐heptadecene (C17, m/z 125), monomethyl terephthalate (m/z 105), and styrene trimer (m/z 91), respectively (Table S5 and Figure S19). [36, 37, 90] Notably, at the respective m/z values, signals were only observed from plastics, confirming no interference from the peptide in the identification of PE (Figure 5c), PET (Figure 5d), PP (Figure 5e), and PS (Figure 5f) NPs. To prevent interference from iron oxide in NP quantification, the recovered MagNanoTrap‐NP complex was treated with 37% HCl at 65°C, leading to iron oxide digestion and aggregate (peptide‐NP complex) formation, prior to Py‐GC/MS analysis (Figure S20). The potential impact of HCl on polymer mass during the treatment process was evaluated (Figure S21). [91] PC, which contains carbonate linkages, and nylon 6 and nylon 6,6, which contain amide bonds, exhibited only minor degradation under the applied conditions. In all cases, more than 98% of the initial polymer mass was retained, indicating that acid‐induced loss is minimal and does not significantly affect subsequent quantification.

To evaluate the 1 L‐scale enrichment performance of MagNanoTrap for trace amounts of PS‐COOH_500 nm_ NPs, optimization comprised determining the ideal quantity of MagNanoTrap beads (Figure 5g), the required incubation time for PS‐COOH_500 nm_ NPs recovery (Figure S22), and the optimal NaCl concentration (Figure S23). Specifically, for 5 µg of spiked PS‐COOH_500 nm_ NPs, an increasing amount of MagNanoTrap beads resulted in improved recovery, with 16 mg of beads achieving a 64.3% recovery (Figure 5g). The general applicability was further investigated by enriching a mixed NP suspension containing 1 µg each of PP, PE, PET, and PS, achieving recoveries above 90% in a 100 mL spiked assay, which demonstrates the effective enrichment of MagNanoTrap and the reliability of the established workflow for NPs quantification. To ensure accurate detection of all NP types, including those less prevalent in environmental water samples, a 1 L spiked assay was performed, yielding recoveries of 65.7%, 67.9%, 67.6%, and 58.7% for PP, PE, PS, and PET, respectively, which is comparable to those obtained using the membrane‐based method [35, 36, 37, 38] (Figure 5h). These results validate the scale‐up to 1 L samples with the employed concentrations down to 1 µg/L, making the MagNanoTrap enrichment technology an attractive tool for NP quantification in natural environments.

### 2.5 NPs Quantification in 1 L Environmental Water Samples

**FIGURE 6:**
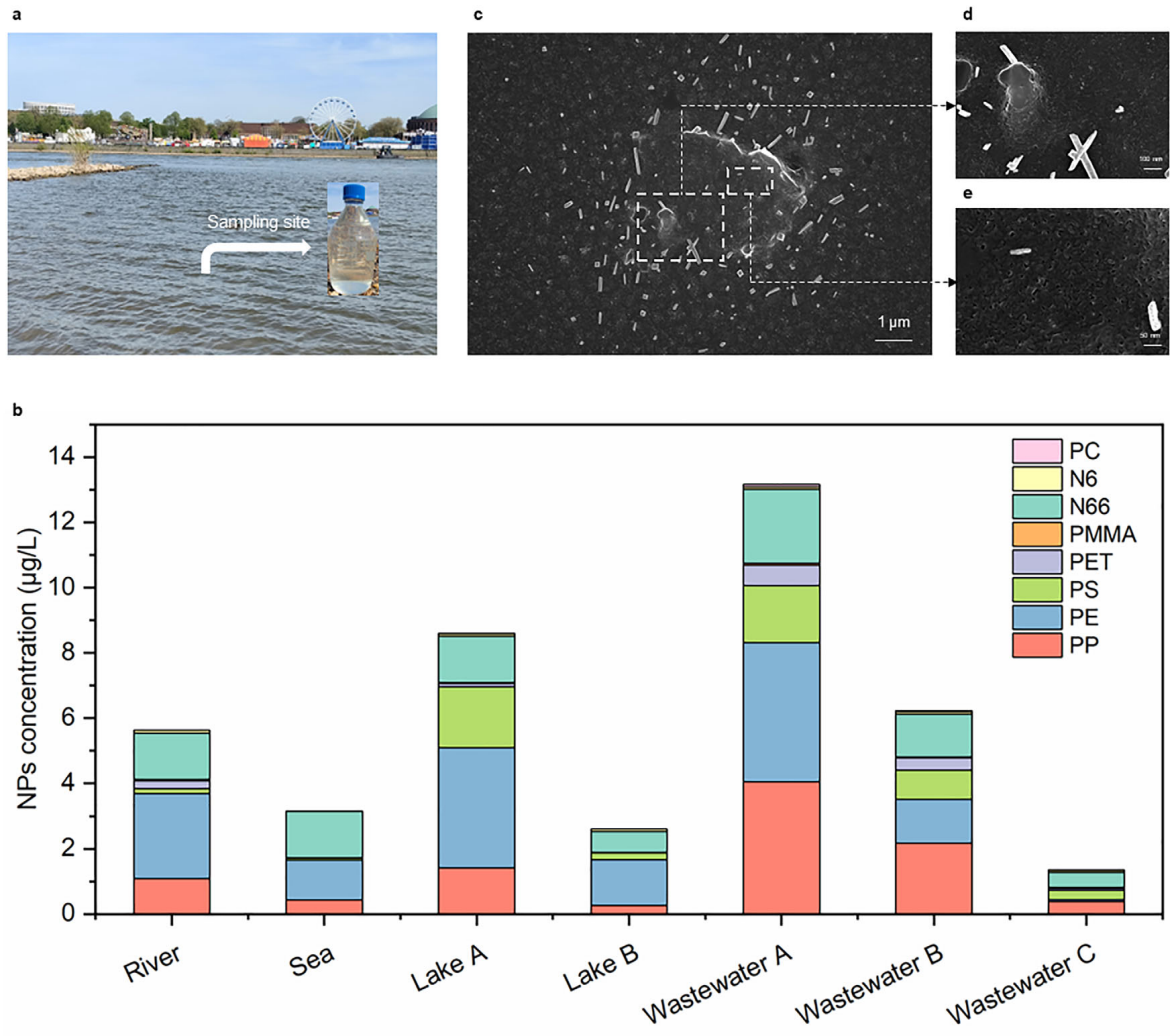
NPs quantification from environmental water samples. (a) Representative sampling site of the river sample in the Rhine River, Düsseldorf, Germany. (b) Quantification and determination of plastic composition in seven types of environmental water samples: river water from the Rhine River, seawater from the coast of Portugal, lake water from the Hangeweiher Park (Lake A) with high human activity, lake water from the Eifel natural reservoir (Lake B) with restricted human activity, wastewater samples obtained from the effluent of the wastewater treatment plant Heiderscheidergrund (Wastewater A), wastewater after sand filtration (Wastewater B) and wastewater after ultrafiltration (Wastewater C). (c) SEM images showing the morphology and size distribution of enriched NPs by MagNanoTrap from wastewater after ultrafiltration (Wastewater C), with insets displaying zoomed areas of NPs, (d) in 100 nm bar, and (e) 50 nm bar.

The application of the MagNanoTrap enrichment technology to quantify NP contaminations in environmental water samples was probed by investigation of seven environmental water samples, including freshwater from the Rhine River (Figure 6a), seawater from the coast of Portugal, lake water from the Hangeweiher Park with high human occurrence, lake water from the Eifel natural reservoir with low human activity, and wastewater from the effluent of the Heiderscheidergrund wastewater treatment plant (WWT). Wastewater samples were also collected after sand filtration and ultrafiltration to determine the effects of advanced treatment processes in WWTs. The selected samples cover fresh water, salt water, and wastewater to determine the application scope of the MagNanoTrap enrichment technology. All environmental water samples were pretreated with H_2_O_2_ to remove organic matter from the NPs' surfaces effectively, [35, 36, 37, 38] ensuring MBP1 binding and accurate quantification by Py‐GCMS (Figure 5a).

In order to determine whether the recovery efficiency is affected by components within the environmental water samples, a set of spiked experiments was performed with all seven environmental water samples by spiking them with PP, PE, PS, and PET NPs (1 µg/L each). The recoveries, ranging from 51.9% to 60.6% for all environmental samples (Table S6), compared to 58.7% to 68.0% in deionized water, highlight the robustness of the MagNanoTrap platform for use in diverse environmental water conditions. With no peak interference from the peptide (Figure S24), four additional types of plastics were also successfully detected and quantified, including PMMA, Nylon 6, Nylon 66, and PC. The detected concentrations of NPs in river water, seawater, lake water in the park, lake water in the natural reservoir, wastewater from effluent, wastewater after sand filtration, and wastewater after ultrafiltration were 5.64 µg/L, 3.16 µg/L, 8.60 µg/L, 2.61 µg/L, 13.16 µg/L, 6.23 µg/L, and 1.35 µg/L, respectively (Figure 6b). The concentrations of NPs detected in the wastewater effluent are consistent with previously published reports, which indicate levels ranging from 5.22 to 12.4 µg/L. In those studies, PP, PE, and nylon 66 were also identified as the dominant NP types in water samples, in agreement with our findings (Figure S25). [35, 38] Interestingly, lake water from the park, influenced by human activities, contained 3.3 times more NPs than lake water from the natural reservoir. For wastewater after different treatments, the plastic content significantly decreased, with ultrafiltration membrane pretreatment removing up to 90% of NPs (Figure 6b). SEM analysis showed that the enriched samples contained NPs with irregular morphologies, including fiber‐like, flake‐like, and ball‐stick structures, particularly in wastewater samples collected after ultrafiltration. Many of these particles were smaller than 20 nm, which can cross the blood–brain barrier and cause serious nerve diseases [28, 29] (Figures 6c and S26).

## 3 Conclusion

In conclusion, a biofunctionalized MagNanoTrap platform that is readily prepared and broadly applicable for various NPs enrichment was developed. Powered by the bifunctional peptide LCI‐DZ‐MBP1 and coupled with Py‐GC/MS analysis, this system offers an innovative approach for water remediation and NPs monitoring. The MagNanoTrap achieved over 96% recovery for PS, PP, PE, and PET NPs, with maximum adsorption capacities of 3.95 ± 0.14 g/g for PS‐COOH_500 nm_, surpassing previously reported adsorbent materials. The adsorption is promoted by salt ions, in which hydrophobic interaction becomes the main driving force for NPs binding, making this platform versatile for the application in all kinds of aqueous solutions. The MagNanoTrap efficiently captured NPs of various sizes from 1 L water samples, achieving comparable recoveries of 65.1 ± 2.9% for PP, PE, PS, and PET NPs. Analysis of seven environmental samples enabled the detection and quantification of eight NP types, including PP, PE, PS, PET, PMMA, PC, Nylon 6, and Nylon 66. The platform's versatility suggests strong potential for use in monitoring polymer contamination in industrial effluents, food matrices, and biological samples. Ultimately, the MagNanoTrap system holds promise as a routine analytical tool for NP contamination monitoring, supporting the goals of a circular polymer economy and contributing to the achievement of SDG 6 on clean water and sanitation.

## Conflicts of Interest

The authors declare no conflicts of interest.

## Supporting information




**Supporting File 1**: anie72039‐sup‐0001‐SuppMat.Pdf.

**Supporting File 2**: anie72039‐sup‐0002‐SuppMovie.Mov.

## Data Availability

The data that support the findings of this study are available from the corresponding author upon reasonable request.
