# Supplementary material for: MagNanoTrap Enrichment Empowers Ultra‐Sensitive Quantification of Mixed Nanoplastic Particles From Environmental Water Samples
Source: Angew Chem Int Ed Engl. 2026 Apr 5;65(20):e22823. doi: 10.1002/anie.202522823 (PMC13159399; doi:10.1002/anie.202522823)
Supplement: Supplementary file 1 — Supporting File 1: anie72039‐sup‐0001‐SuppMat.Pdf. [file ANIE-65-e22823-s002.pdf]

Supplementary information

MagNanoTrap Enrichment Empowers Ultra-sensitive Quantification of Mixed Nanoplastic Particles from Environmental Water Samples

Maochao Mao,[a] Marian Bienstein,[a] Francisca Contreras,[a] Dong Wang,[a] Lilin Feng,[a] and Ulrich Schwaneberg*[a]

[a]Lehrstuhl für Biotechnologie, RWTH Aachen University, Worringerweg 3, 52074 Aachen, Germany

*Corresponding author, E-mail address: u.schwaneberg@biotec.rwth-aachen.de

Contents

1. Materials 3
2. Protein expression and purification 3
3. Preparation of MagNanoTrap beads 3
4. Characterization 3
5. Preparation of PP and PE nanoplastics 4
6. Preparation of PET nanoplastics 4
7. 200 μL-scale Enrichment 4
8. Kinetic and isotherm models 4
9. Zn^{2+} adsorption 5
10. Environmental water sample collection 5
11. 1 L-scale enrichment 5
12. Nanoplastics quantification by Py-GCMS 6
13. Nanoplastics visualization by SEM 6
14. Results 7
15. References 40

1. Materials

Fe_3O_4 nanoparticles in water dispersion were purchased from Particular Materials SRL (Italy). PS nanoparticles with different charges and sizes were purchased from Polysciences Europe GmbH (Germany). LD-PE and PET powder were purchased from Goodfellow Cambridge Ltd (Germany). PP granule was purchased from Sigma-Aldrich (Merck, Germany). Strep-Tactin®XT DY-649 was purchased from IBA Lifesciences GmbH (Germany). The microplastics calibration standard-low set was purchased from Frontier Lab (Japan). Sodium chloride, hydrogen peroxide, zincon sodium salt, xylene, and other laboratory-grade chemicals were purchased from Sigma-Aldrich (Merck, Germany) or AppliChem (Germany) unless specified.

2. Protein expression and purification

The DNA sequences encoding LCI-strep-DZ, strep-DZ-MBP1, and LCI-strep-DZ-MBP1 (Supplementary Table 7) were synthesized and inserted into the pET-28a (+) vector by GenScript Biotech (Netherlands). The plasmids were transformed into competent *E. coli* BL21(DE3) cells for protein expression. Following overnight incubation at 37 °C in a shaker, the pre-culture was used to inoculate 50 mL of Lysogeny Broth (LB) medium (supplemented with 50 μg/mL kanamycin) and cultured at 37 °C with continuous shaking at 200 rpm. When the OD_{600} reached 0.4-0.6, the main cultures were induced with 0.1 mM IPTG and further incubated at 18 °C, 200 rpm overnight. Cells harvested by centrifugation were resuspended in buffer A (50 mM Tris-HCl, pH 8, and 300 mM NaCl) and lysed by sonication. The supernatant collected after centrifugation was applied to a gravity column packed with Strep-Tactin®XT 4Flow® resin, pre-equilibrated with buffer A. After washing with 10 column volumes of buffer A, strep-tagged proteins were eluted with buffer B (50 mM Tris-HCl, pH 8, 300 mM NaCl, and 2.5 mM desthiobiotin). Buffer exchange of the eluted protein into the final stock buffer C (50 mM Bicine-NaOH, pH 9) was performed with a PD-10 Desalting Column (Cytiva). The whole purification and buffer exchange process was done at 4 °C, and the protein concentration was measured by UV absorption at 280 nm.

3. Preparation of MagNanoTrap beads

The purified protein was mixed with Fe_3O_4 nanoparticles in a functionalization buffer (50 mM Bicine/NaOH, pH 9) by shaking at 1200 rpm in glass vials (Screw neck vial ND 8, TH. GEYER GmbH & Co. KG, Germany). Magnetic extraction was then used to wash the beads with water, after which they were resuspended in water for subsequent use.

4. Characterization

The fluorescence microscope (BX51, Olympus) was used to check the binding of peptides on material surfaces. The fluorescence-activated cell sorting (FACS; MoFlo Astrios EQ, Beckman Coulter) was used to analyze the functionalization efficiency of MagNanoTrap beads. The dynamic light scattering (Zetasizer, Malvern Panalytical) was used to measure the size distribution of nanoparticles. The thermogravimetric analysis (TGA; STA6000, PerkinElmer) was used to examine the mass loss of samples during temperature increase. The X-ray photoelectron spectroscopy (XPS; AXIS supra +, Kratos Analytical) and Fourier-transform infrared spectroscopy (FTIR; Nicolet iS20, Thermo Scientific) were used to study the surface chemistry of magnetic beads. An electrospray ionization time-of-flight mass spectrometry (ESI-TOF MS; Agilent) was used to assess the successful functionalization of magnetic beads by a bifunctional peptide. The SQUID magnetometer (MPMS-XL, Quantum Design) was used

to study the magnetic properties of the beads. A scanning electron microscope (SEM; SU9000, Hitachi) was used to study the morphology of the samples. A transmission electron microscopy (TEM; SU9000, Hitachi) was used to observe the inner structure of particles.

5. Preparation of PP and PE nanoplastics

PP and PE nanoparticles were synthesized as described by Faeze *et al.*[1] with modifications. Briefly, 10 mg of PP granules or LD-PE powder were dissolved in 5 mL of xylene at 110 °C in a glass beaker on a heater for 10 minutes until the solids were completely dissolved. With vigorous stirring, 20 mL of icy deionized water was rapidly added to emulsify the mixture, followed by immediate water bath sonication for 30 minutes. Once the PP/PE particles were suspended in water, Whatman Grade 1 qualitative filter paper was used to filter out the larger particles. The filtration process was repeated one more time after mild magnetic stirring for 2 hours at room temperature to obtain the nanoparticle dispersion in water.

6. Preparation of PET nanoplastics

PET nanoparticles were prepared according to a reported protocol[2]. Briefly, 10 mg of PET powder was dissolved in 2 mL 1,1,1,3,3,3-hexafluoro-2-propanol at room temperature for 1 h. The PET solution was dropped into 20 mL of deionized water with vigorous magnetic stirring. The organic solvent was removed from the particle suspension by continuous mild stirring for 2 hours at room temperature, followed by paper filtration (Whatman Grade 1) to remove the precipitated particles, and the nanoparticle-containing filtrate is ready for later characterization.

7. 200 µL-scale Enrichment

Enrichment on a 200 µL scale was done in the glass vial and characterized by UV absorbance measurement (CLARIOstar, BMG Labtech). Specifically, the prepared MagNanoTrap beads were mixed with 0.2 g/L of PS NPs and 150 mM NaCl in a water solution at a total volume of 200 µL. Followed by shaking at 1200 rpm for 20 min and magnetic extraction, 100 µL of supernatant was transferred into a 96-well plate for absorbance characterization. The concentration was obtained according to the UV-vis standard curves, and the NPs recovery rate (R_1) was calculated using equation (1),

$$R_1 = \left(1 - \frac{C_t}{C_0}\right) * 100\% \tag{1}$$

where C_t represents the NPs concentration in the supernatant after magnetic extraction and C_0 is the concentration of the original NPs water dispersion. The adsorption uptake q (g/g) was calculated using equation (2),

$$q = \frac{C_0 - C_t}{C_m} \tag{2}$$

where C_m is the concentration of MagNanoTrap beads. Each experiment was done in triplicate.

8. Kinetic and isotherm models

The adsorption kinetic process was described by two adsorption models in this study, including Pseudo-first-order (3) and Pseudo-second-order (4)[3].

$$q_t = q_e(1 - e^{-k_1 t}) \tag{3}$$

$$q_t = \frac{k_2 q_e^2 t}{1 + k_2 q_e t} \tag{4}$$

The q_t and q_e are the amounts of NPs adsorbed per unit mass of MagNanoTrap beads at time, *t,* and at equilibrium, respectively; k_1 and k_2 are adsorption rate constants for the Pseudo-first-order and Pseudo-second-order, respectively.

The adsorption isotherm was described by two adsorption models in this study, including the Freundlich model (5) and the Langmuir model (6)[4].

$$q_e = K_F C_e^{1/n} \quad (5)$$

$$q_e = \frac{q_m K_L C_e}{1+K_L C_e} \quad (6)$$

The K_F (L/g) and K_L (L/g) are the isotherm constants for the Freundlich and Langmuir models, respectively; C_e (g/L) is the concentration of adsorbate, PS NPs; q_m and q_e are the adsorption capacity at maximum and equilibrium, respectively.

9. Zn^{2+} adsorption

The concentration of Zn^{2+} was measured using a zincon assay, with the absorbance recorded at 620 nm via UV-vis spectrophotometry. Briefly, 0.3 g/L MagNanoTrap beads and 0.2 g/L PS-COOH 500 nm NPs were mixed with 20 mM Zn^{2+}, either individually or together, and shaken at 1200 rpm for 10 minutes. After centrifugation at 10,000 rpm for 1 minute, the supernatant was diluted 80-fold and mixed with 1 mM zincon solution for concentration analysis.

10. Environmental water sample collection

The lake water samples in the natural reservoir were collected from Eifel National Park (Monschau, Germany). The lake water samples from the park with human activities were collected from Hangeweiher (Aachen, Germany). The river water samples were collected from the Rhine River (Düsseldorf, Germany). The seawater from a depth of 50 m off the coast of Portugal was ordered from Mrutzek Meeres-Aquaristik GmbH (Germany). The wastewater was collected from the effluent of the wastewater treatment plant SIDEN, located in Heiderscheidergrund (Luxembourg). Wastewater samples from the efflux, after sand filtration and ultrafiltration using a crossflow membrane from Boll & Kirch, were supplied by APATEQ (Luxembourg). All water samples were stored in glass bottles with PP caps at 4 °C, except for the seawater, which was contained in a PE bottle.

11. 1 L-scale enrichment

For method optimization, deionized water spiked with NPs was used, whereas environmental water samples required a pretreatment process before extraction. In detail, each 1 L water sample underwent vacuum filtration through 1 μm glass fiber filters (47 mm) on a stainless steel filtration unit to remove large aggregates (Fig. 5a). The filtrate was collected in a 5 L glass flask, followed by oxidative digestion with 100 mL of 30% H_2O_2 at 60 °C for 48 hours in an orbital incubator shaker to remove the organic matter (Minitron, INFORS, Switzerland)[5-6]. After digestion, the samples were cooled down to room temperature, mixed with 16 mg of MagNanoTrap beads and 1 M NaCl, and shaken at 200 rpm for 2 h to facilitate adsorption. The water hybrid was poured into a 500 mL glass beaker for magnetic extraction of NPs. The extracted mixture was resuspended in 5 mL of deionized water and transferred into a 1.5 mL glass vial for magnetic extraction to further reduce the volume. Subsequently, the MagNanoTrap-NPs complex was treated with 37% HCl at 65 °C for 30 min, followed by

filtration using a 10 kDa centrifugal filter (VWR, UK), after which the aggregates were resuspended in methanol and transferred into a Py-GCMS sampling cup for later analysis. The recovery rate (R_2) of the spiked assay was calculated using equation (7),

$$R_2 = \frac{m_2 - m_1}{m_0} * 100\% \quad (7)$$

where m_0, m_1, and m_2 were the spiked amounts of NPs, and the detected amounts of NPs before and after spiking, respectively.

12. Nanoplastics quantification by Py-GCMS

Quantification of eight types of NPs, including PP, PE, PS, PET, Nylon 6, Nylon 66, PC, PMMA, and PVC, was performed using a Multi-Shot Micro-furnace Pyrolyzer (EGA/PY-3030D) with an Auto-Shot Sampler (AS-2020E) from Frontier Laboratories (Japan), coupled to an Agilent GC/MS (8890 GC System and 7000D GC/TQ, USA). The Py-GC/MS analysis parameters were chosen according to previously reported methods (Supplementary Table 4)[5-8]. To identify and quantify plastics in environmental samples, specific indicator compounds/ions are necessary and summarized (Supplementary Table 5). For the calibration curve preparation, varying amounts (1.0, 3.0, 5.0, 7.0, and 9.0 mg) of the standard mixture (MPs-SiO_2 provided by Frontier Laboratories) were weighed into the pyrolysis cup (80 µL) prior to analysis. The Py-GC/MS method was validated by assessing linearity (R^2), limit of detection (LOD), and limit of quantification (LOQ), with these parameters calculated for each plastic polymer using the corresponding calibration curves and applying equations (8) and (9), respectively[5-8]:

$$LOD = 3x\frac{\alpha}{S} \quad (8)$$

$$LOQ = 10x\frac{\alpha}{S} \quad (9)$$

where α represents the residual standard deviation of the regression equation, while S denotes the slope of the calibration curve.

13. Nanoplastics visualization by SEM

Following NP enrichment from each environmental water sample in a 1.5 mL glass vial, the samples were treated with 200 µL of 6 M HCl and shaken at room temperature overnight to dissolve the iron oxide. After acid digestion, the samples were analyzed using SEM.

14. Results

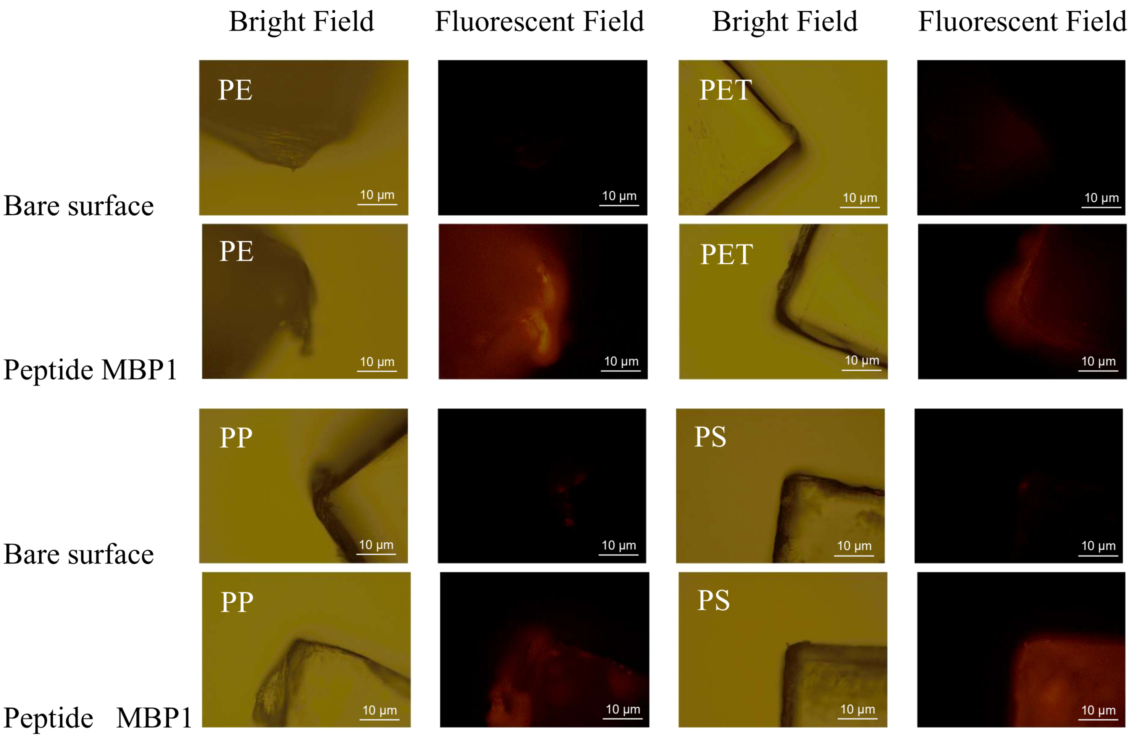

Figure S1. Qualitative evaluation of MBP1 binding toward different plastic surfaces. The fluorescent dye Strep-Tactin®XT DY-649 was used to label the strep-DZ-MBP1 peptide specifically. The detector gain value was adjusted for each polymer type to suppress background autofluorescence from the bare plastic surface. Under these optimized settings, fluorescence originating from peptide binding could be clearly distinguished.

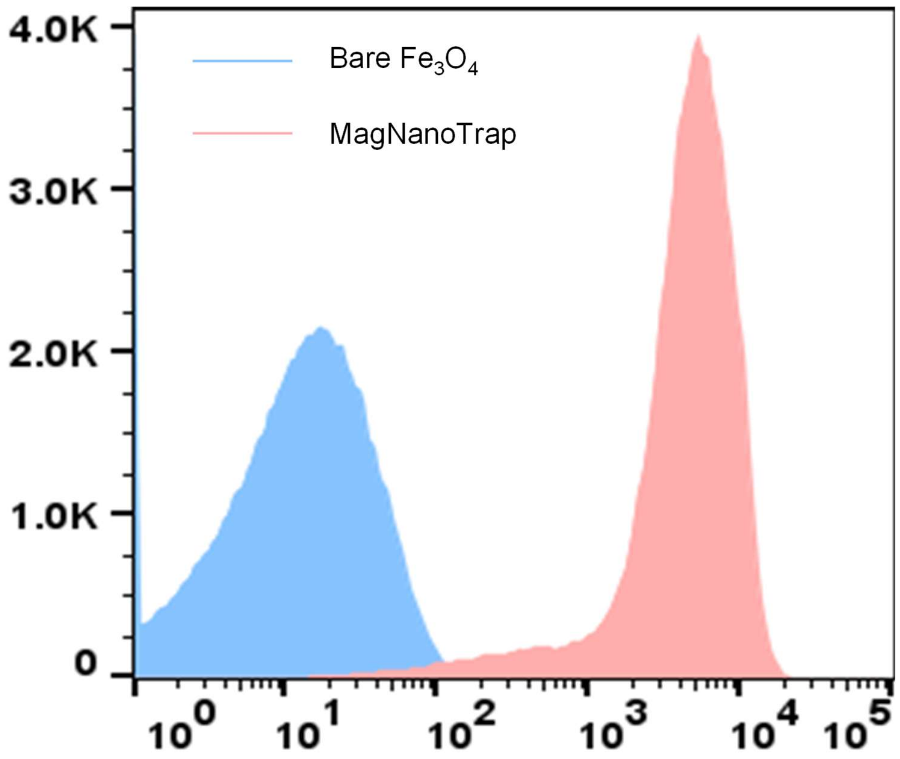

Figure S2. FACS analysis of MagNanoTrap and bare iron oxide. The fluorescent dye Strep-Tactin®XT DY-649 was used to specifically label LCI-strep-DZ-MBP1 peptide binding on the iron oxide bead surface. An obvious fluorescence shift was observed in peptide-decorated MagNanoTrap beads, indicating the successful decoration of SPIONs by the peptide.

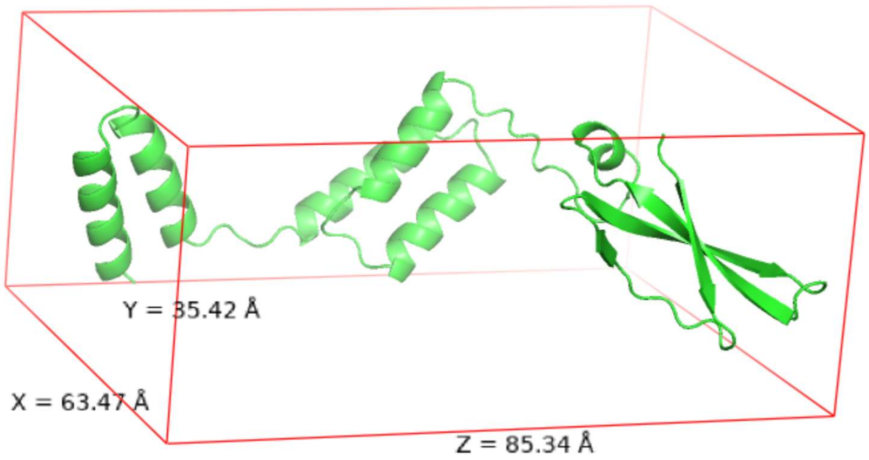

Figure S3. Predicted size of LCI-strep-DZ-MBP1 peptide by PyMOL. Peptide was shown with a height of 8.5 nm.

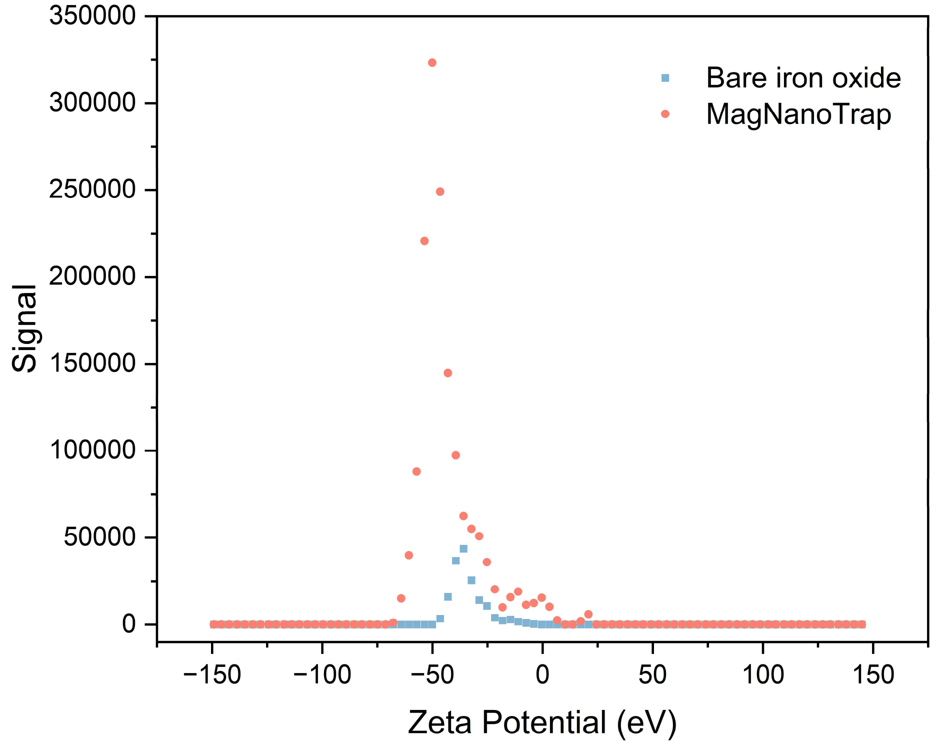

Figure S4. Zeta potential measurement of MagNanoTrap and bare iron oxide. The beads were negatively charged before and after functionalization.

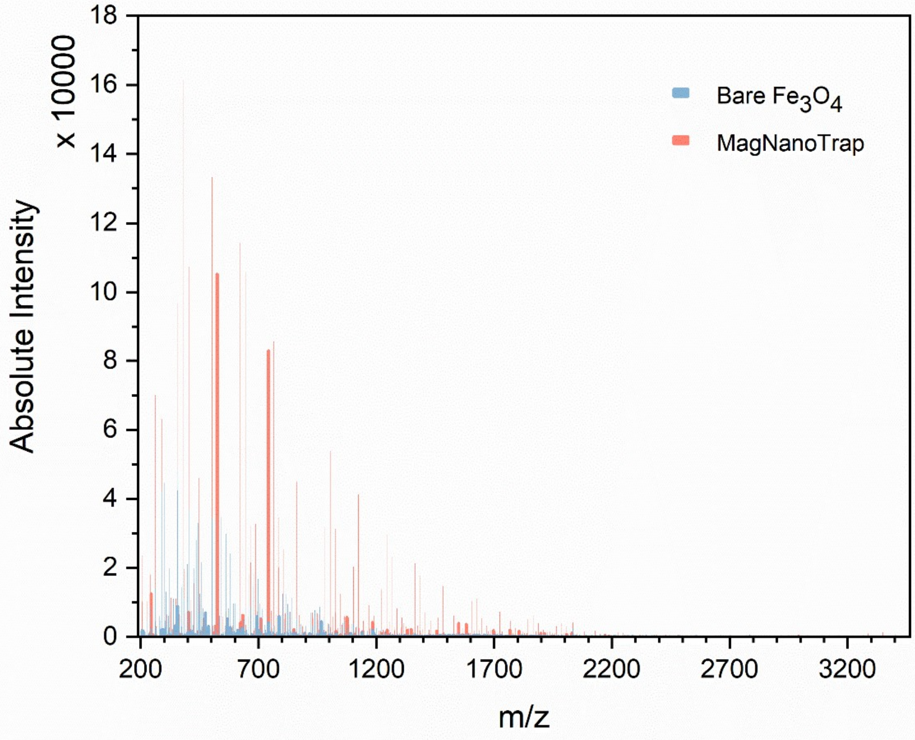

Figure S5. ESI-MS analysis of MagNanoTrap and bare iron oxide. An obvious signal from peptide LCI-DZ-MBP1 was observed in MagNanoTrap beads, indicating the successful decoration of SPIONs by the peptide.

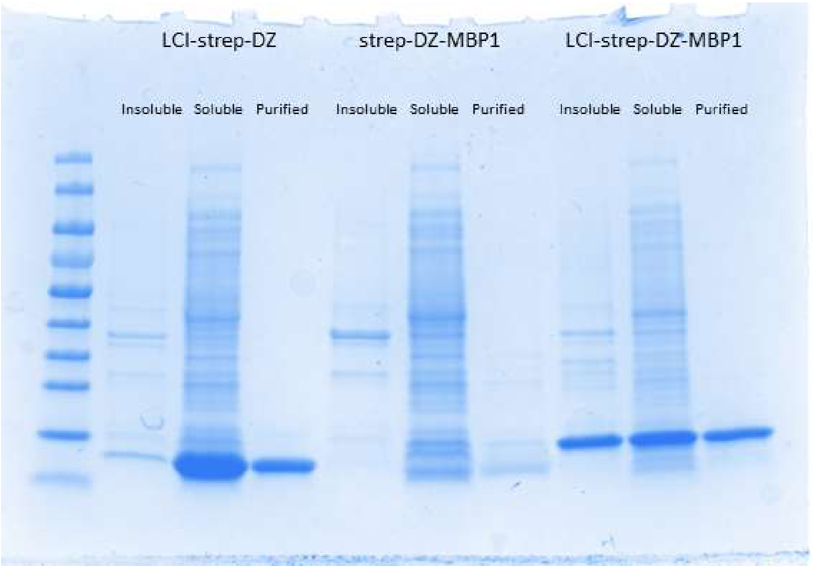

Figure S6. SDS-PAGE of peptide LCI-strep-DZ, strep-DZ-MBP1, and LCI-strep-DZ-MBP1. Peptides with strep-tag were successfully purified, and LCI-DZ-MBP1 matches 17.49 kDa.

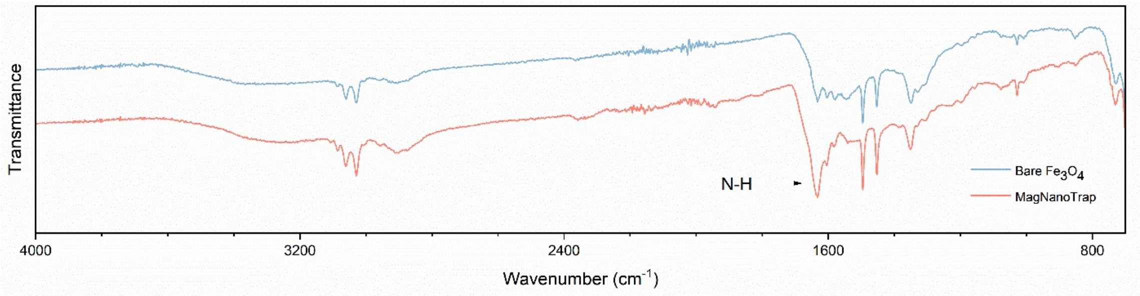

Figure S7. FTIR analysis of MagNanoTrap and bare iron oxide. An increasing signal of N-H bond was observed in MagNanoTrap beads, indicating the successful decoration of SPIONs by the peptide.

Table S1. Calibration curves of NPs with different sizes and charges characterized by absorbance measurement.

Plastic type	Wavelength	Linear range (g/L)	Calibration equation	Linearity (R^2)
PS-COOH $_{100\ nm}$	350 nm	0.00625-0.4	y=1.2586x-0.0053	0.9999
PS-NH_2 $_{100\ nm}$	340 nm	0.00625-0.2	y=1.6657x-0.0018	0.9998
PS-COOH $_{500\ nm}$	460 nm	0.003125-0.2	y=5.4455x+0.0041	0.9998
PS-COOH $_{1000\ nm}$	550 nm	0.003125-0.2	y=4.6207x-0.0120	0.9990
Self-made PET	340 nm	0.00625-0.2	Y=0,7029x+0,117	0,9984

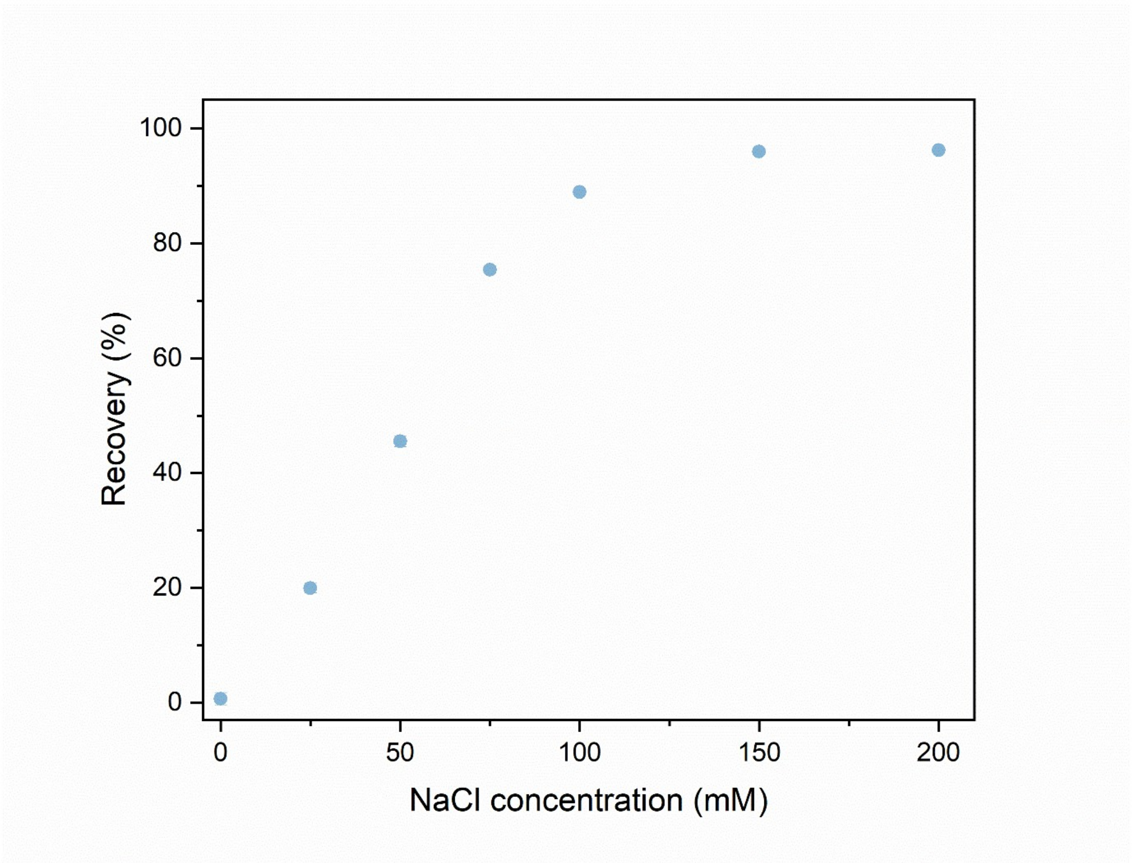

Figure S8. Enrichment performance at different NaCl concentrations, n = 3, mean ± SD. Enrichment of PS-$COOH_{500\ nm}$ was initiated with the addition of NaCl, with 150 mM of NaCl reaching the saturated recovery.

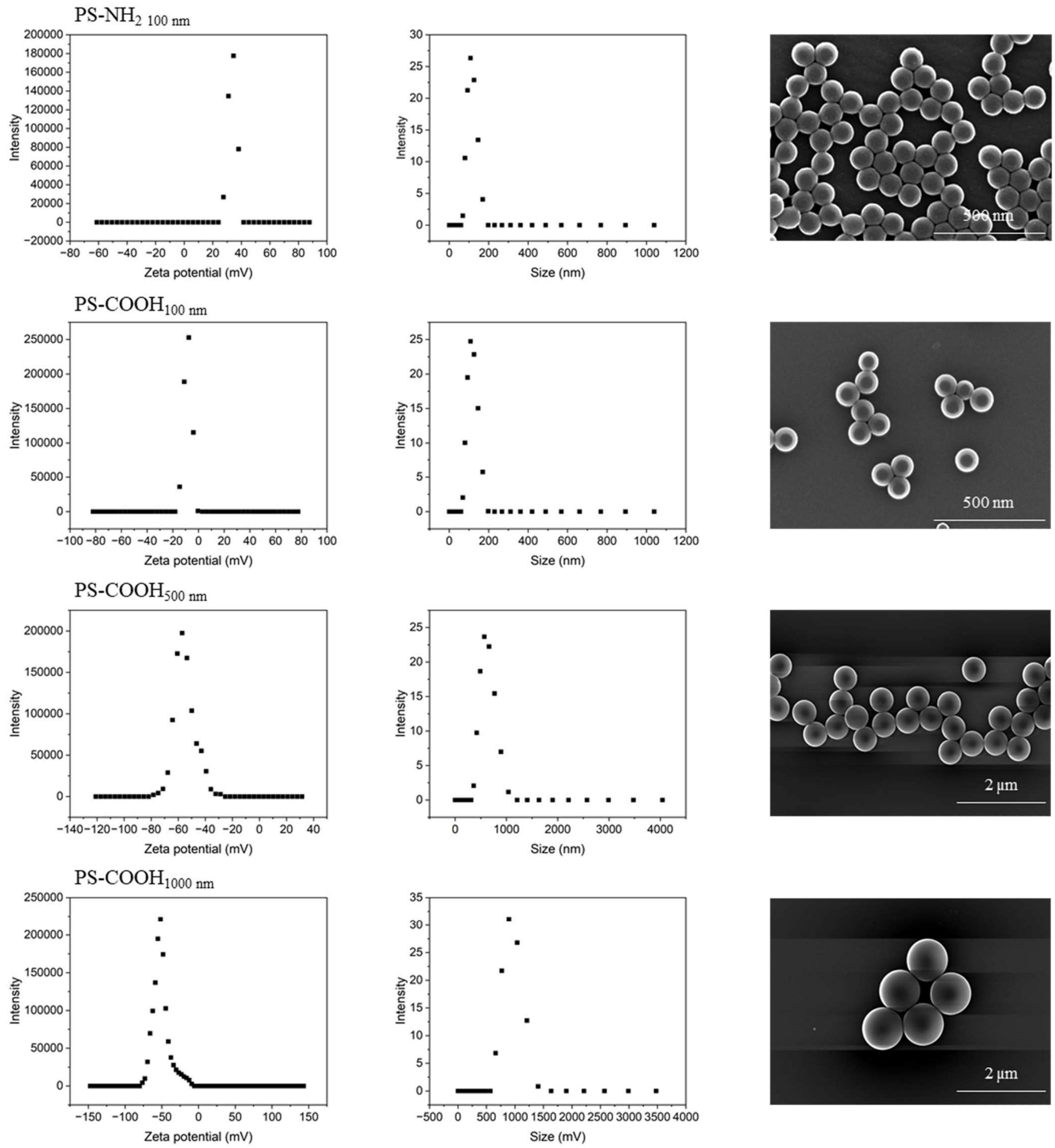

Figure S9. NPs characterization. DLS (left), Zeta potential (middle), and SEM (right) of commercial PS NPs.

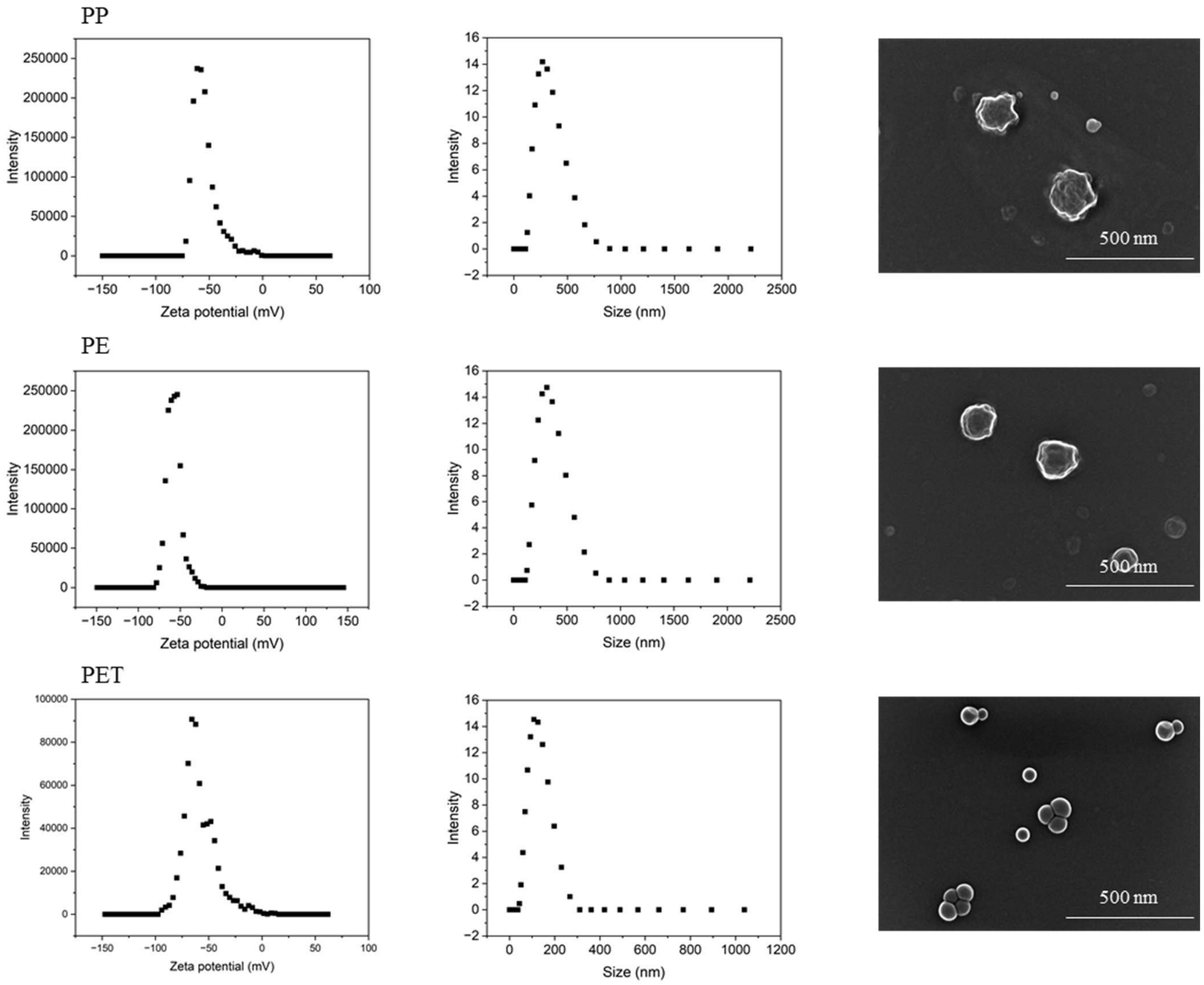

Figure S10. NPs characterization. DLS (left), Zeta potential (middle), and SEM (right) of self-made PP, PE, and PET NPs.

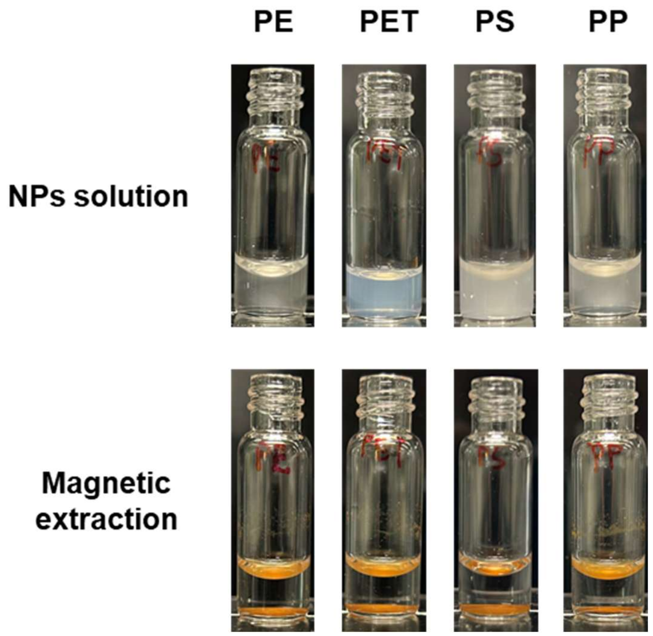

Figure S11. Visualization of 0.2 g/L dispersion of PE, PET, PS, and PP NPs before (top) and after magnetic extraction (bottom) by MagNanoTrap beads.

Table S2. Parameters of the adsorption kinetics equation of MagNanoTrap against PS-COOH$_{500\ nm}$ at different NaCl concentrations, n = 3, mean ± SD.

	Pseudo-first-order model			Pseudo-second-order model		
NaCl concentration	q_e (g/g)	k_1 (min^{-1})	R^2	q_e (g/g)	k_2 (g/(g·min))	R^2
100 mM	0.56 ± 0.02	0.70 ± 0.11	0.9709	0.63 ± 0.01	1.36 ± 0.16	0.9934
150 mM	0.60 ± 0.02	1.23 ± 0.18	0.9623	0.66 ± 0.01	2.44 ± 0.27	0.9918
200 mM	0.61 ± 0.02	1.79 ± 0.22	0.9711	0.64 ± 0.01	4.04 ± 0.29	0.9962

Table S3. Parameters of the adsorption isotherm equation of MagNanoTrap against PS-$COOH_{500\ nm}$ at different NaCl concentrations, n = 3, mean ± SD.

	Freundlich model			Langmuir model		
NaCl concentration	1/n	K_F (L/g)	R^2	q_m (g/g)	K_L (L/g)	R^2
100 mM	0.54 ± 0.03	1.33 ± 0.03	0.9855	3.05 ± 0.08	0.85 ± 0.05	0.9985
150 mM	0.40 ± 0.04	2.13 ± 0.05	0.9587	3.80 ± 0.09	1.40 ± 0.09	0.9981
200 mM	0.33 ± 0.04	2.53 ± 0.04	0.9331	3.95 ± 0.14	1.85 ± 0.21	0.9970

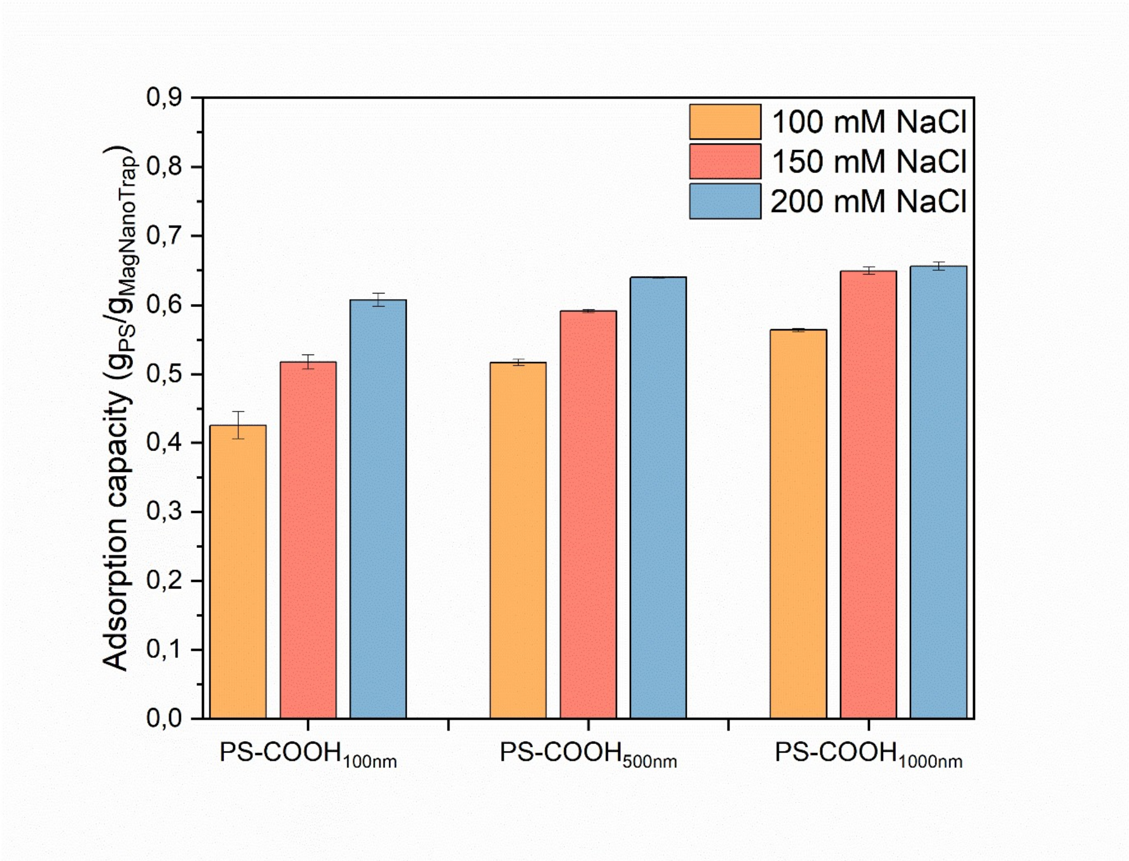

Figure S12. Adsorption capacity of PS-COOH NPs with different sizes at different NaCl concentrations, n = 3, mean ± SD. A higher NaCl concentration enhances adsorption capacity, and larger PS-COOH NPs exhibit greater adsorption efficiency.

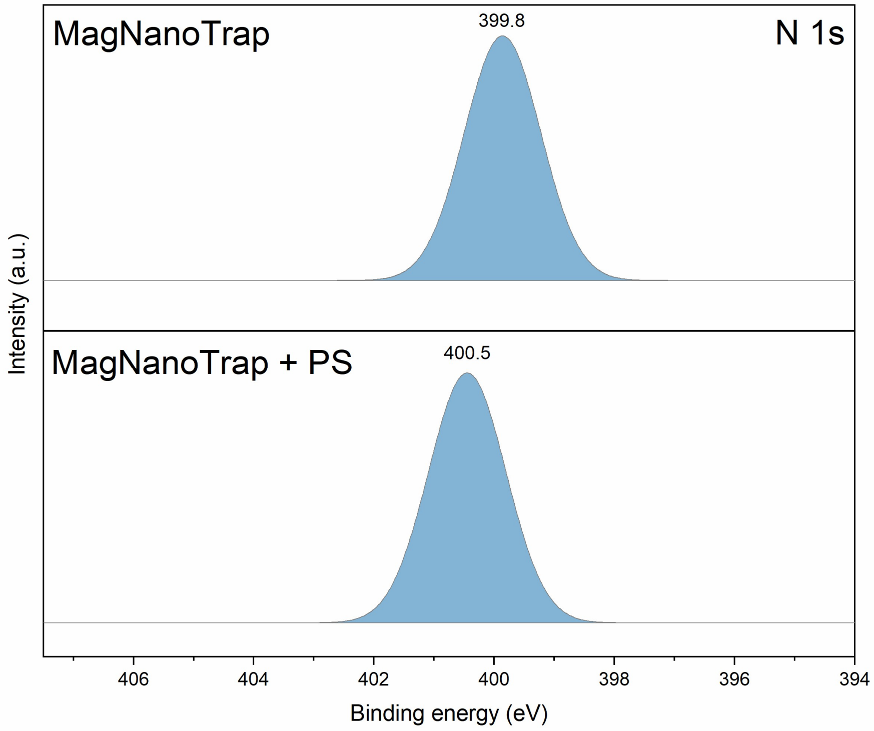

Figure S13. XPS spectra (N 1s) of MagNanoTrap (top) and MagNanoTrap-PS conjugate (bottom).

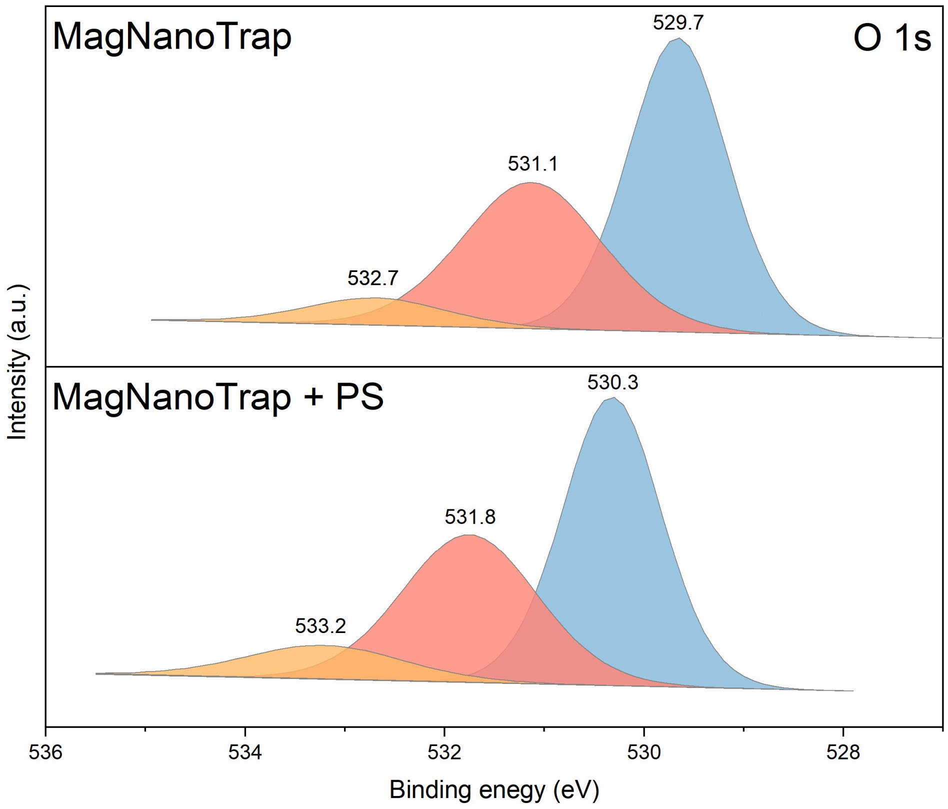

Figure S14. XPS spectra (O 1s) of MagNanoTrap (top) and MagNanoTrap-PS conjugate (bottom).

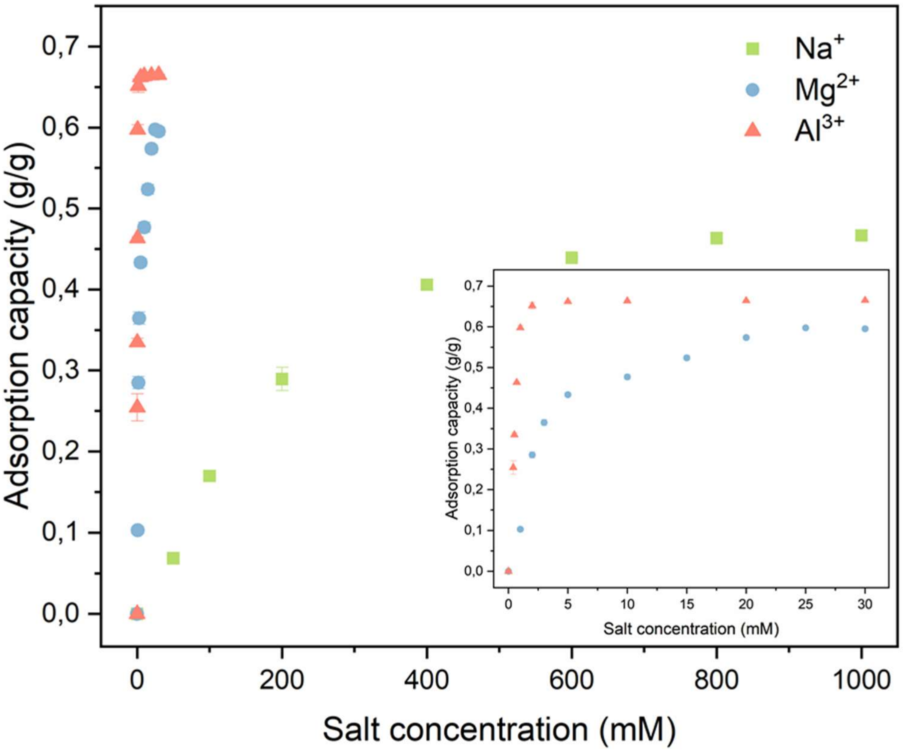

Figure S15. Adsorption capacity of PS-COOH$_{500\ nm}$ NPs at different NaCl, $MgCl_2$, and $AlCl_3$ concentrations, n = 3, mean ± SD.

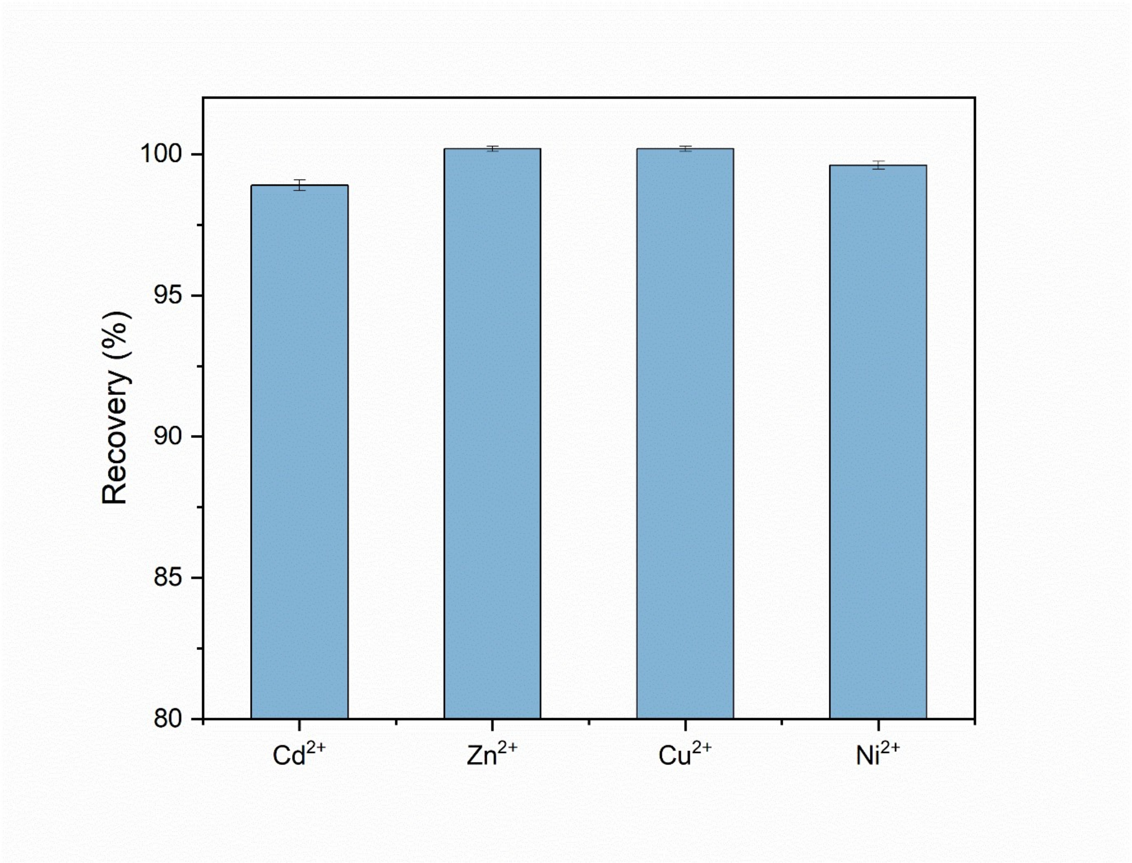

Figure S16. Recovery of PS-COOH $_{500\ nm}$ NPs with the addition of heavy metals, n = 3, mean ± SD. With the addition of heavy metals instead of NaCl, the recovery exceeds 99%, confirming that the presence of heavy metals does not interfere with the enrichment process.

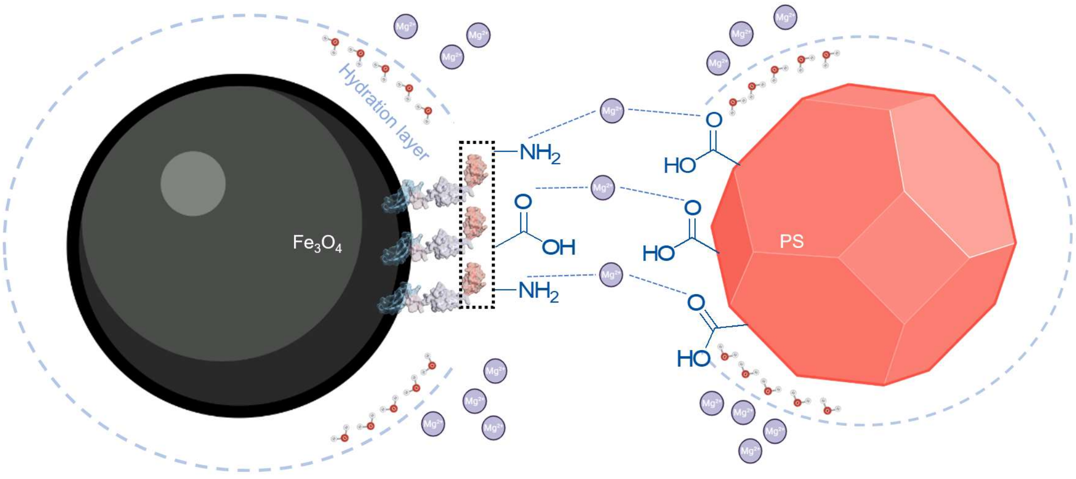

Figure S17. Scheme illustrating the Mg^{2+} effects on NPs adsorption by MagNanoTrap beads. Mg^{2+} bridges PS-COOH NPs and MagNanoTrap beads through Lewis acid-base interactions.

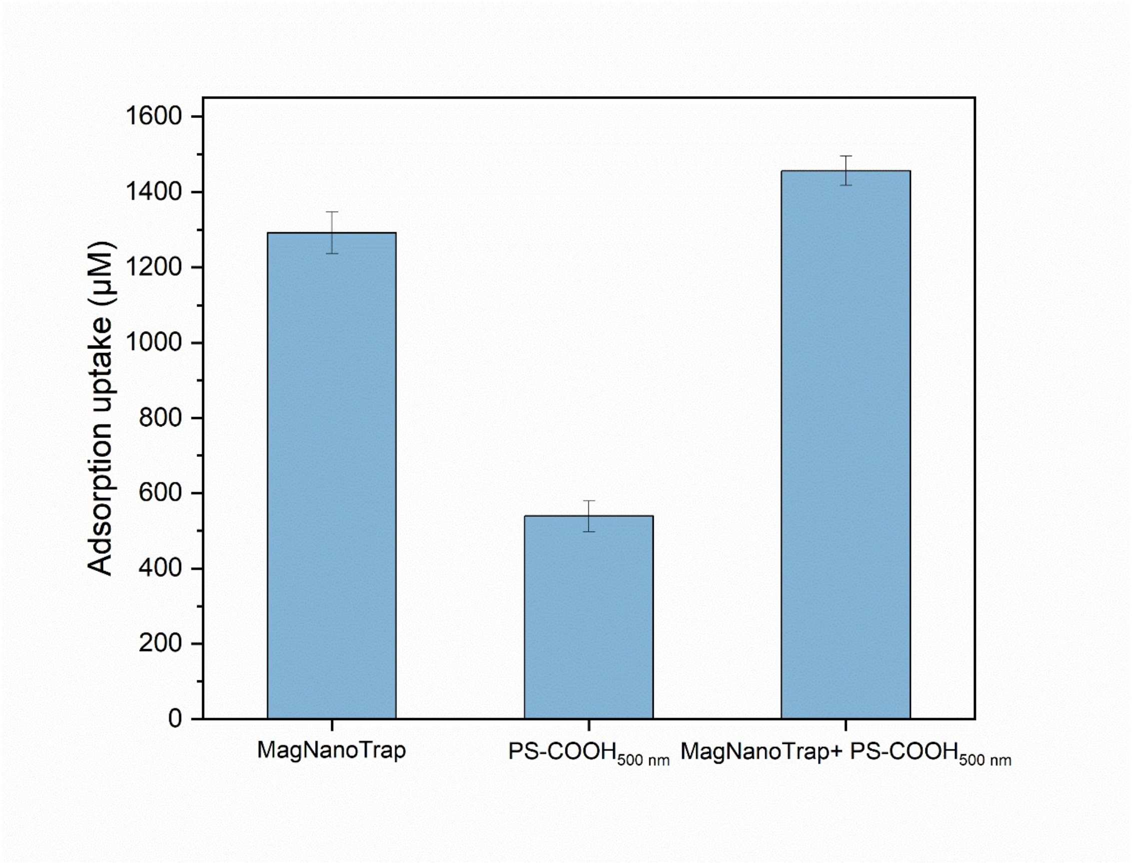

Figure S18. Adsorption uptake of Zn^{2+} by MagNanoTrap and PS-COOH$_{500\ nm}$, both individually and in combination, n = 3, mean ± SD. PS-COOH$_{500\ nm}$ NPs incubated at a Zn^{2+} concentration of 20 mM together with MagNanoTrap beads reached the total Zn^{2+} adsorption at only 1456.4 ± 38.7 µM, less than the sum of the individual adsorption capacities (1292.3 ± 55.5 µM for MagNanoTrap beads and 538.5 ± 40.7 µM for PS-COOH$_{500\ nm}$ NPs), suggesting that Zn^{2+} ions mediate the interaction between PS-COOH$_{500\ nm}$ NPs and MagNanoTrap beads.

Table S4. Conditions for single-shot Py-GC/MS measurements.

**Micro-furnace pyrolyzer**	Frontier EGA/PY-3030D
Carrier gas	Helium
Pyrolysis time	0.3 min
Pyrolysis temperature	590 °C
Interface temperature	300 °C
**Gas chromatograph**	Agilent 8890 GC System
Column	Frontier Ultra Alloy 5; 30 m, 0.25 mm I.D., 0.25 µm film thickness
Column flow	1 mL/min
Injector port temperature	300 °C
Temperature program	40 °C (2 min) ⟶ 320 °C (20 °C/min, 14 min)
Split mode	30:1
**Mass spectrometer**	Agilent 7000D GC/TQ
Ionization energy	70 eV
Scan range	10-550 m/z
Ion source temperature	230 °C

Table S5. Characteristic components and calibration functions of the eight analyzed nanoplastic polymer types.

Polymer	Indicator compound	Indicator ions (m/z)	Calibration functions	Linearity (R^2)	LOD (ng)	LOQ (ng)
PE	1-Heptadecene (C17)	125	y=9639.058x+17634.639	0.994	58.62	177.64
PP	2,4,6-Dimethyl-1-heptene	126	y=19794.827x+5293.108	0.996	200.50	607.57
PS	Styrene trimer	91	y=3620170.055x+91565.638	0.999	60.67	183.84
PET	Monomethyl terephthalate	105	y=624495.860x-6139.633	0.996	42.76	129.60
PMMA	Methyl methacrylate	100	y=2774172.354x-865.402	0.999	1.30	3.95
PC	p-Isopropenylphenol	134	y=3438297.015x+23987.357	0.987	0.81	2.45
Nylon 6	*N*-(5-cyanopentyl)hex-5-enamide	154	y=2690993.850x+133328.500	0.990	13.12	39.76
Nylon 66	Cyclopentanone	84	y=697560.697x+168218.346	0.990	145.26	440.18

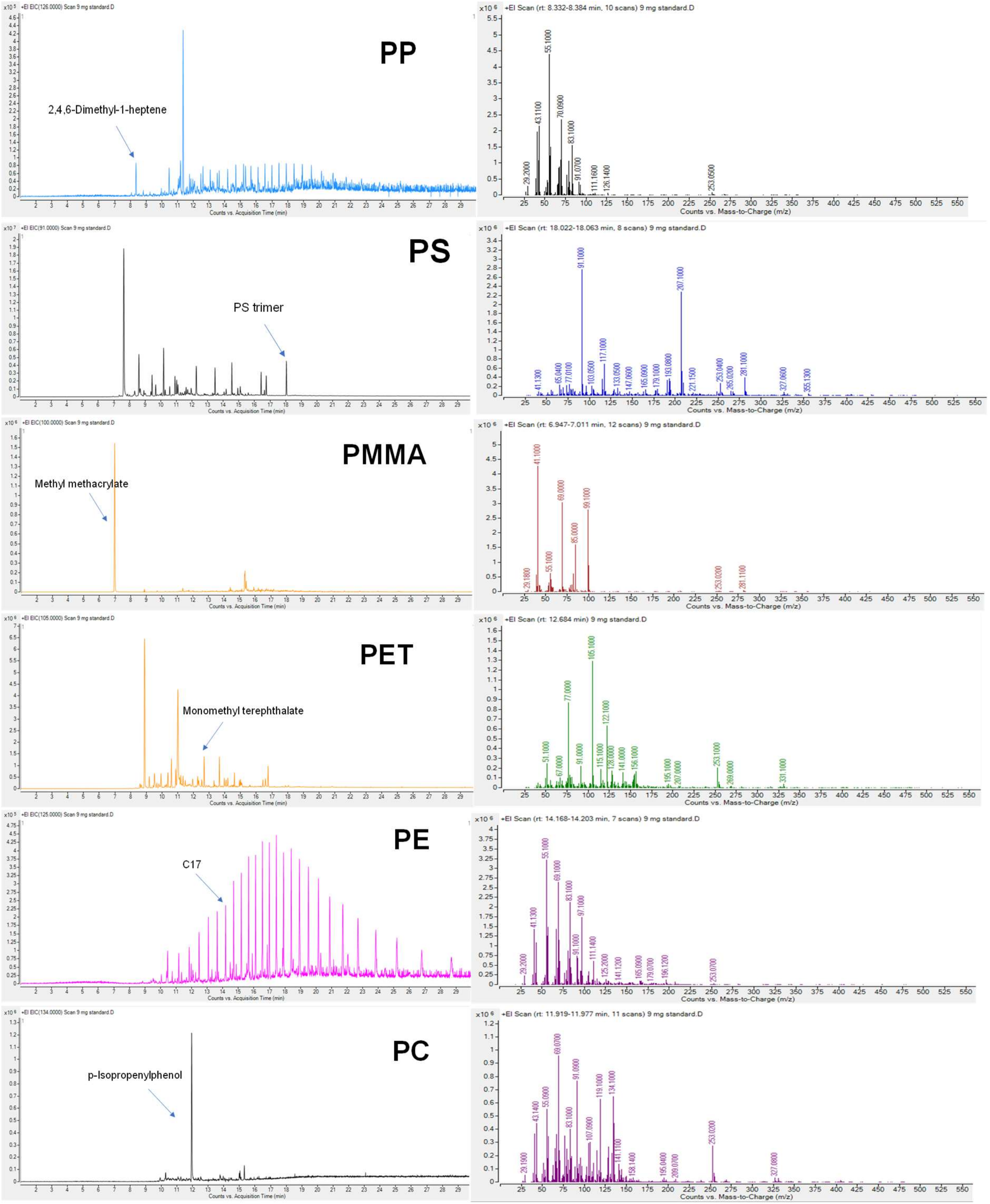
PP
2,4,6-Dimethyl-1-heptene
PS
PS trimer
PMMA
Methyl methacrylate
PET
Monomethyl terephthalate
PE
C17
PC
p-Isopropenylphenol

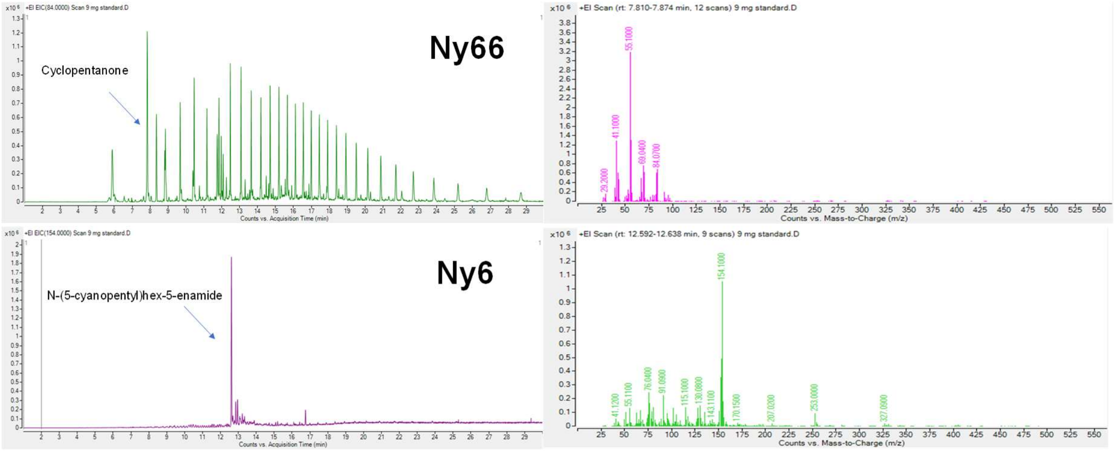

Figure S19. GC/MS spectrum chromatography of each plastic type, including PP, PS, PMMA, PET, PE, PC, Ny6, and Ny66. These eight types of plastics can be successfully detected by Py-GC/MS.

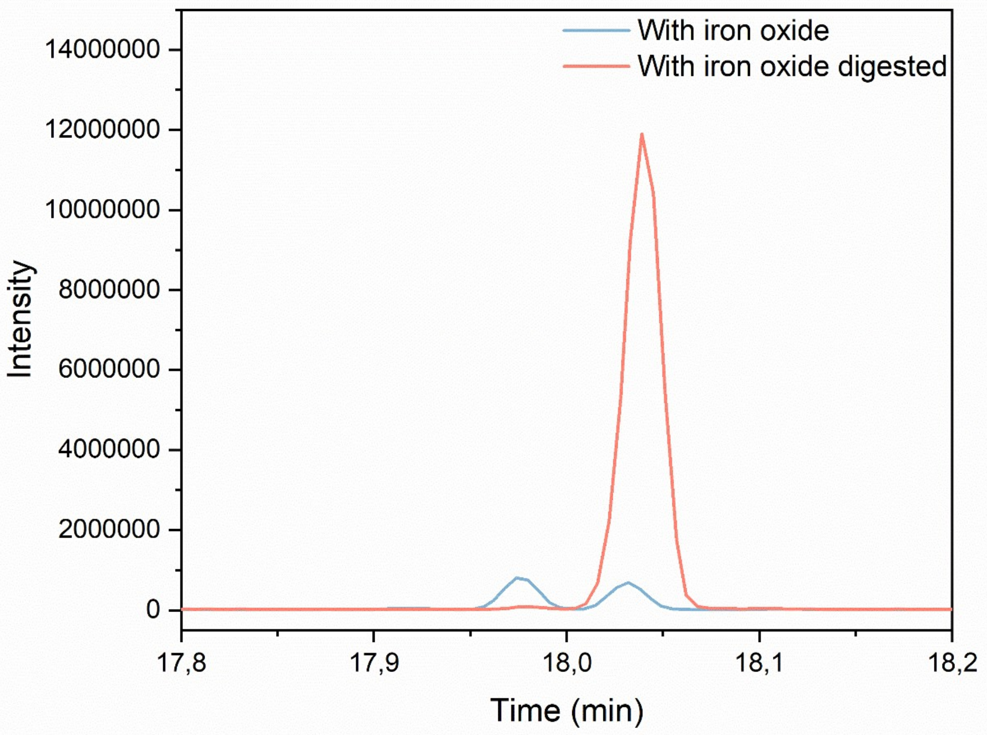

Figure S20. Py-GC/MS chromatography of 5 µg PS NPs captured by MagNanoTrap beads. Without iron oxide digestion, the specific peak (m/z 91) for the PS trimer is divided into two peaks, and the signal shows a dramatic decrease, suggesting the importance of removing iron oxide before plastics analysis by Py-GC/MS.

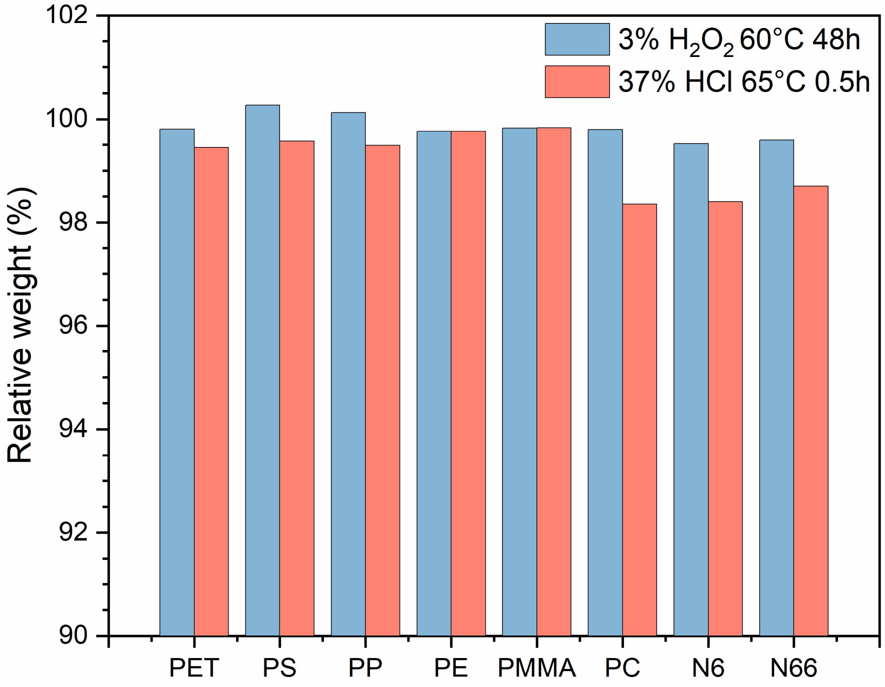

Figure S21. Relative weight of plastics after treatment with H_2O_2 and HCl under enrichment conditions quantified by Py-GC/MS. H_2O_2 showed no significant effect on polymer weight loss. For PC, which contains carbonate linkages (–O–COO–), and for nylon 6 and nylon 6,6, which contain amide bonds (–CONH–), treatment with 37% HCl at 65 °C for 30 min led to only slight degradation. In all cases, the remaining mass was still >98%, indicating that any acid-induced loss is minimal and is not expected to substantially affect subsequent quantification.

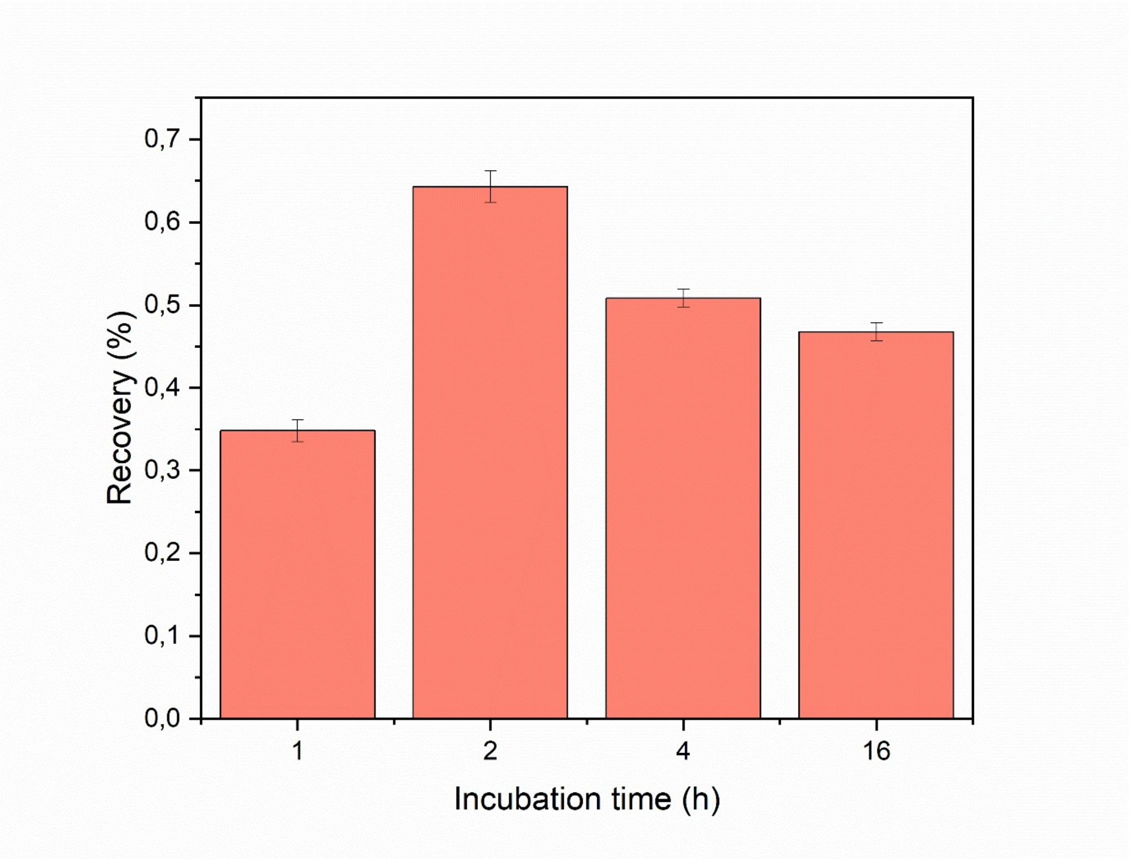

Figure S22. Recovery of PS-COOH$_{500\ nm}$ NPs (5 µg spiked in 1 L deionized water) at different shaking times, with 1 M NaCl and 16 mg MagNanoTrap beads, n = 3, mean ± SD.

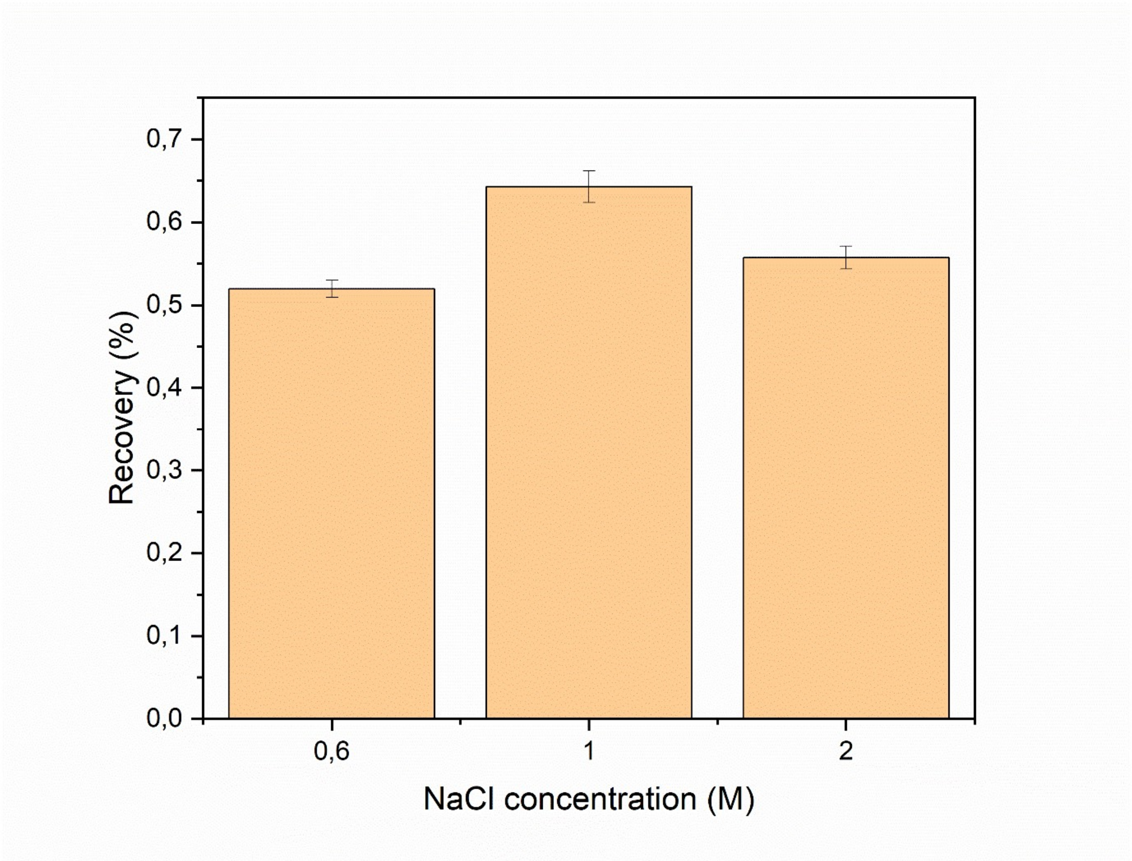

Figure S23. Recovery of PS-COOH$_{500\ nm}$ NPs (5 µg spiked in 1 L deionized water) at different NaCl concentrations, with 2 h shaking time and 16 mg MagNanoTrap beads, n = 3, mean ± SD.

Table S6. Determination of the concentration and spiked recovery of NPs in environmental waters by the proposed method. For each type of environmental water sample, 1 µg of PP, PE, PS, and PET NPs was also spiked into 1 L of the corresponding water samples to study the recovery.

	PP		PE		PS		PET		PMMA	Ny66	N6	PC	SUM
Sample	Detected (µg/L)	Recovery (%)	Detected (µg/L)	Recovery (%)	Detected (µg/L)	Recovery (%)	Detected (µg/L)	Recovery (%)	Detected (µg/L)	Detected (µg/L)	Detected (µg/L)	Detected (µg/L)	Detected (µg/L)
ddH_2O	ND		ND		ND		ND		ND	ND	ND	ND	ND
Spiked	0,657	65,75	0,679	67,95	0,676	67,65	0,587	58,66					
River	1,089		2,609		0,148		0,239		0,035	1,416	0,090	0,008	5,636
Spiked	1,630	54,09	3,198	58,82	0,725	57,68	0,762	52,21					
Lake in the park	1,410		3,684		1,863		0,103		0,033	1,415	0,078	0,012	8,598
Spiked	1,969	55,86	4,270	58,60	2,441	57,88	0,623	51,99					
Lake in the natural reservoir	0,259		1,402		0,211		ND		0,019	0,641	0,072	0,001	2,605
Spiked	0,839	57,99	2,003	60,09	0,810	59,88	0,555	55,48					
Sea	0,433		1,220		0,061		ND		0,013	1,412	0,018	0,005	3,160
Spiked	1,039	60,61	1,783	56,33	0,603	54,21	0,543	54,35					
Wastewater	4,046		4,265		1,746		0,624		0,062	2,267	0,084	0,069	13,161
Spiked	4,620	57,43	4,840	57,51	2,327	58,02	1,149	52,50					
Wastewater after sand filtration	2,176		1,340		0,893		0,367		0,034	1,318	0,077	0,021	6,226
Spiked	2,762	58,60	1,901	56,09	1,470	57,72	0,886	51,92					
Wastewater after ultrafiltration	0,388		0,062		0,272		0,069		0,022	0,468	0,052	0,018	1,350
Spiked	0,955	56,75	0,647	58,47	0,832	56,00	0,593	52,42					

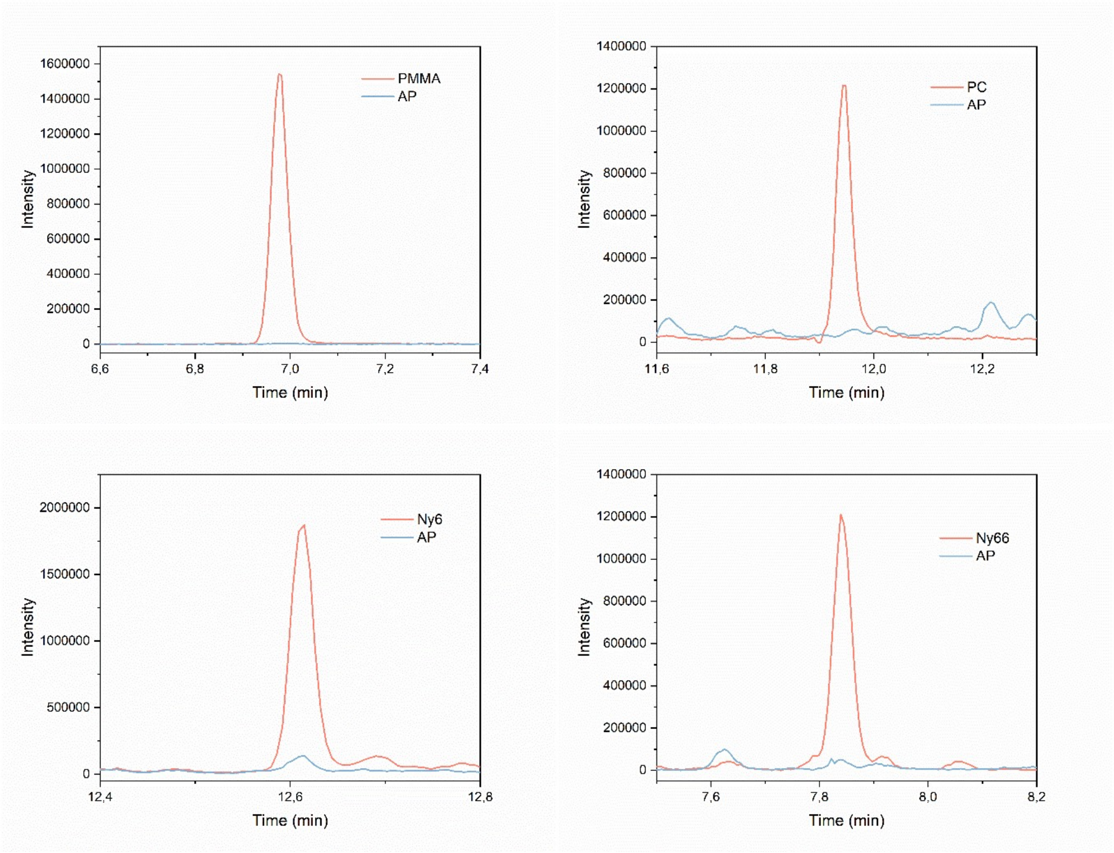

Figure S24. Chromatograms with an extracted m/z of PMMA (m/z 100), PC (m/z 134), Ny6 (m/z 154), and Ny66 (m/z 84) from both mixed plastics and peptides. It shows no interference from the peptide LCI-DZ-MBP1 at the respective peaks for PMMA, PC, Ny6, and Ny 66 analysis by Py-GC/MS.

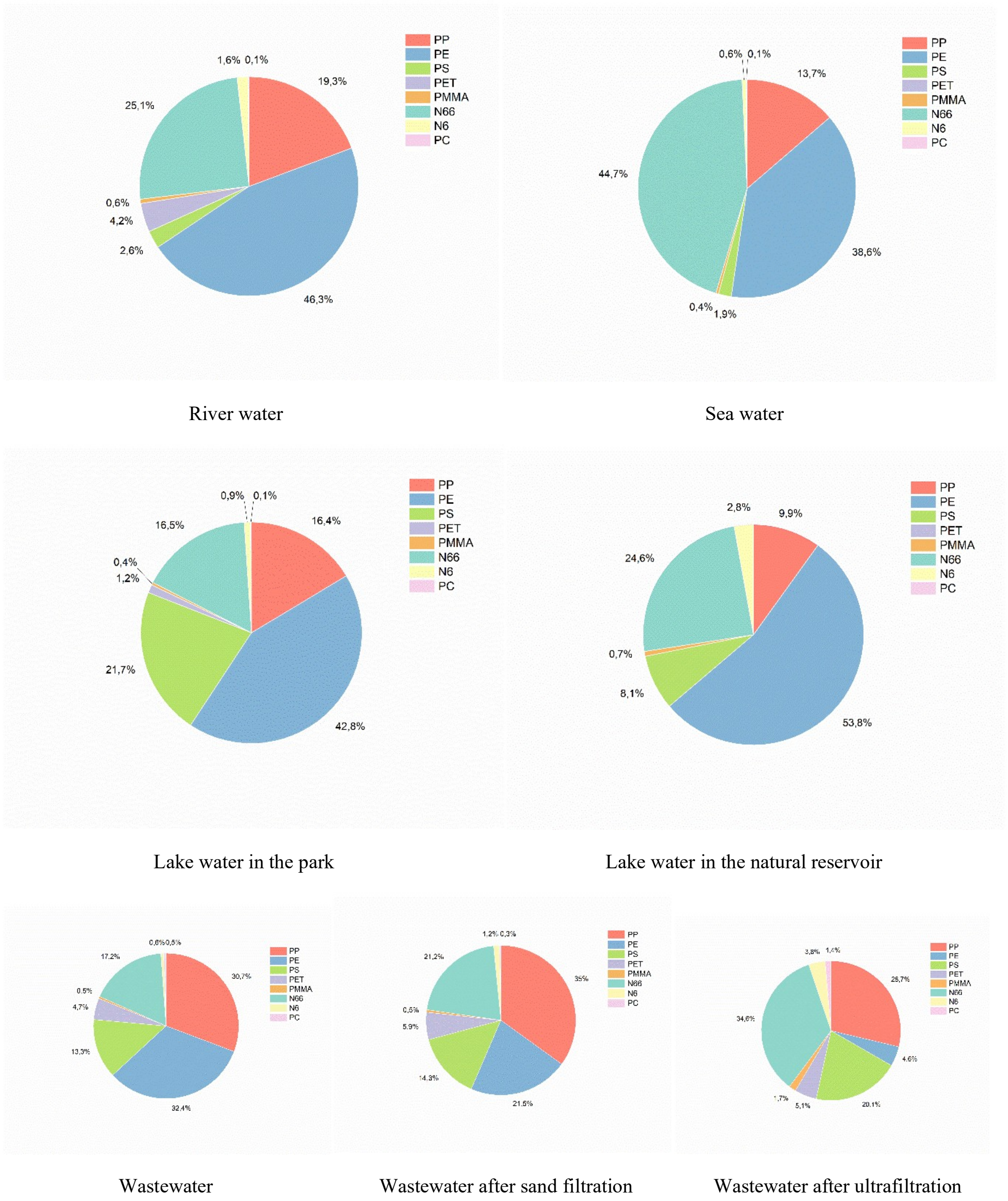

Figure S25. Plastic distribution in detected environmental water samples. NPs from the environmental water are enriched by the MagNanoTrap platform and quantified by Py-GC/MS. PP, PE, and Ny 66 are the dominant plastic types for environmental water samples.

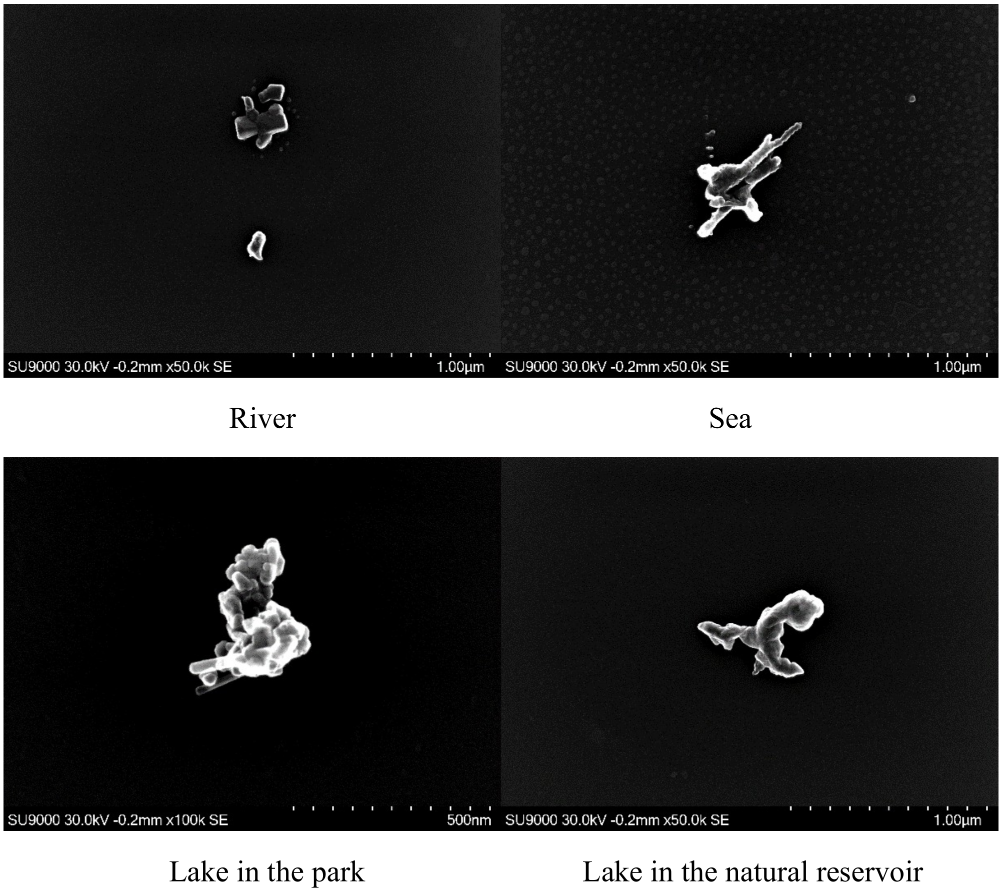

Figure S26. SEM images of NPs in the environmental water samples. NPs from environmental water samples enriched by the MagNanoTrap platform are of irregular morphologies.

Table S7. Protein sequence information used in this manuscript.

Protein	Amino acid sequence
LCI-strep-DZ	MAIKLVQSPNGNFAASFVLDGTKWIFKSKYYDSSKGYWV GIYEVWDRKSAWSHPQFEKADNKFNKEQQNAFYEILHLP NLNEEQRNGFIQSLKDDPSQSANLLAEAKKLNDAQAPK
strep-DZ-MBP1	MSAWSHPQFEKADNKFNKEQQNAFYEILHLPNLNEEQRN GFIQSLKDDPSQSANLLAEAKKLNDAQAPKRSGRGECRRQ CLRRHEGQPWETQECMRRCRRRG
LCI-strep-DZ-MBP1	MAIKLVQSPNGNFAASFVLDGTKWIFKSKYYDSSKGYWV GIYEVWDRKSAWSHPQFEKADNKFNKEQQNAFYEILHLP NLNEEQRNGFIQSLKDDPSQSANLLAEAKKLNDAQAPKRS GRGECRRQCLRRHEGQPWETQECMRRCRRRG

15. References

[1] F. H. Zokaei, S. Gharavi, E. Asgarani, M. Zarrabi, M. Soudi, Z. Moosavi-nejad, *Biologia* **2023**, *78*, 179-186.

[2] K. Vogel, R. Wei, L. Pfaff, D. Breite, H. Al-Fathi, C. Ortmann, I. Estrela-Lopis, T. Venus, A. Schulze, H. Harms, U. T. Bornscheuer, T. Maskow, *Sci. Total Environ.* **2021**, *773*, 145111.

[3] J. Wang, X. Guo, *J. Hazard. Mater.* **2020**, *390*, 122156.

[4] M. A. Al-Ghouti, D. A. Da'ana, *J. Hazard. Mater.* **2020**, *393*, 122383.

[5] E. D. Okoffo, K. V. Thomas, *J. Hazard. Mater.* **2024**, *464*, 133013.

[6] E. D. Okoffo, K. V. Thomas, *Water Res.* **2024**, *254*, 121397.

[7] Y. Xu, Q. Ou, X. Wang, F. Hou, P. Li, J. P. van der Hoek, G. Liu, *Environ. Sci. Technol.* **2023**, *57*, 3114-3123.

[8] Y. Xu, Q. Ou, M. Jiao, G. Liu, J. P. van der Hoek, *Environ. Sci. Technol.* **2022**, *56*, 4988-4997.
